# Epitomics: IgG-epitome decoding of E6, E7 and L1 proteins from oncogenic human papillomavirus type 58

**DOI:** 10.1038/srep34686

**Published:** 2016-10-06

**Authors:** Wan-Xiang Xu, Jian Wang, Hai-Ping Tang, Ya-Ping He, Qian-Xi Zhu, Satish K. Gupta, Shao-Hua Gu, Qiang Huang, Chao-Neng Ji, Ling-Feng Liu, Gui-Ling Li, Cong-Jian Xu, Yi Xie

**Affiliations:** 1 Division of Reproductive Immunology, Key Lab of Reproduction Regulation of NPFPC, Shanghai Institute of Planned Parenthood Research, Fudan University, Shanghai 200032, China; 2 Reproductive Cell Biology Laboratory, National Institute of Immunology, New Delhi 110067, India; 3 State Key Laboratory of Genetic Engineering, Institute of Genetics, School of Life Sciences, Fudan University, Shanghai 200433, China; 4 Division of Clinical Research, Fred Hutchinson Cancer Research Center, Seattle, WA 98109, USA; 5 Shanghai Key Laboratory of Female Reproductive Endocrine Related Diseases, Hospital of Obstetrics and Gynecology, Fudan University, Shanghai 200011, China

## Abstract

To enable rational multi-epitope vaccine and diagnostic antigen design, it is imperative to delineate complete IgG-epitome of the protein. Here, we describe results of IgG-epitome decoding of three proteins from high-risk (HR-) oncogenic human papillomavirus type 58 (HPV58). To reveal their entire epitomes, employing peptide biosynthetic approach, 30 precise linear B-cell epitopes (BCEs) were mapped on E6, E7 and L1 proteins using rabbits antisera to the respective recombinant proteins. Using sequence alignment based on BCE minimal motif, the specificity and conservativeness of each mapped BCE were delineated mainly among known HR-HPVs, including finding 3 broadly antibody cross-reactive BCEs of L1 that each covers almost all HR-HPVs. Western blots revealed that 13 of the 18 BCEs within L1-epitome were recognized by murine antisera to HPV58 virus-like particles, suggesting that these are antibody accessible BCEs. Also, a highly conserved epitope (YGD/XTL) of E6 was found to exist only in known common HR-HPVs, which could be used as the first peptide reference marker for judging HR-HPVs. Altogether, this study provides systemic and exhaustive information on linear BCEs of HR-HPV58 that will facilitate development of novel multi-epitope diagnostic reagents/chips for testing viral antibodies and ‘universal’ preventive HPV peptide vaccine based on L1 conserved BCEs.

Cervical cancer is the third most common cancer among women and it has been linked with persistent infection of high-risk (HR) oncogenic human papillomaviruses (HPVs)^1^, which will result in an estimated 530 000 new cases and 275 000 deaths from cervical cancer worldwide every year^2^. Since peptides based on linear B cell epitopes (BCEs) on a target protein(s) could be used as serodiagnostic agents and candidates for synthetic peptide vaccine, epitope mapping is crucial for both basic and applied research in virology, including HPV^3^,^4^,^5^,^6^,^7^,^8^,^9^. However, due to limitation of methodology, it has been impossible to reveal whole nonconformational IgG-epitome for a target protein when using either human antisera, or rabbit/mouse polyclonal antibodies (pAbs) generated against denatured recombinant (r-) or native proteins. Hence, a limited number of BCE peptides or only few type specific, common and neutralizing BCE peptides have been identified^10^,^11^,^12^,^13^. Thus, in the fields of immunology and virology, it has been technically challenging to decode IgG-recognized whole epitome consisting of each BCE fine motif and to reveal all type-restricted and/or type-conserved BCEs among homologous proteins of HPVs, including determining antibody accessible and/or neutralizing/protecting BCEs.

In previous studies, we developed an improved biosynthetic peptide method for epitope mapping, which is simple, cheap, reliable and with adaptable merits, in particular being able to identify antibody-binding minimal motif of each mapped longer antigenic peptide when using pAbs^14^,^15^. Based on the fact that HR-HPV type 58 (HPV58, Accession Number D90400) is more prevalent in China and Eastern Asia^16^,^17^, and is one of the 12 HR-HPVs defined by the World Health Organization (WHO)^18^, we selected it as the target to reveal IgG-epitomes of three key proteins (oncogenic E6 and E7 as well as major capsid L1 proteins) in the present study.

The aims of this study are: i) revealing all linear BCEs of E6, E7 and L1 of HR-HPV58 using antisera of rabbits raised against respective *E. coli*-expressed recombinant proteins (r-proteins); ii) determining conservativeness or specificity of each mapped BCE among their homologous proteins; iii) analyzing the antibody accessibility of all mapped BCEs; and iv) confirming the result of mapped L1-epitome using murine antisera to HPV58-L1 virus-like particles (VLPs). These investigations have provided novel and comprehensive data about linear BCEs on E6, E7, and L1 proteins of HPV58 as well as their unique distribution profile. In addition, we also found three broadly antibody cross-reactive BCEs of L1 and one E6 marker that only existed in HR-HPVs.

## Results

### E6-epitome

E6 is an oncogenic protein of HR-HPVs for cervical cancer and one of the major target antigens or its antigenic peptide(s) to detect serum antibody during HPV infection^4^,^8^,^9^. Hence, it is essential to delineate all potential BCEs on E6 protein [149 amino acid (aa)] of HPV58. To achieve this, E6 corresponding to HPV58 was expressed in *E. coli* and pAbs generated against r-E6 in rabbits (Supplementary Figures S1A and S2A). Using biosynthetic approach, a set of overlapping 15mer peptides (P1–P23, P23 is 17mer) with an overlap of 9 aa corresponding to the full-length sequence of E6 protein were expressed as fusion protein with truncated glutathione S-transferase (GST) carrier protein (188 aa in length; named GST188) in *E. coli*. In addition, forty one 8mer peptides (P24–P64) with an overlap of 7 aa residues each, spanning the reactive 15/17mer peptides, were also expressed as fusion protein with GST188 in *E. coli*.

**Figure 1:**
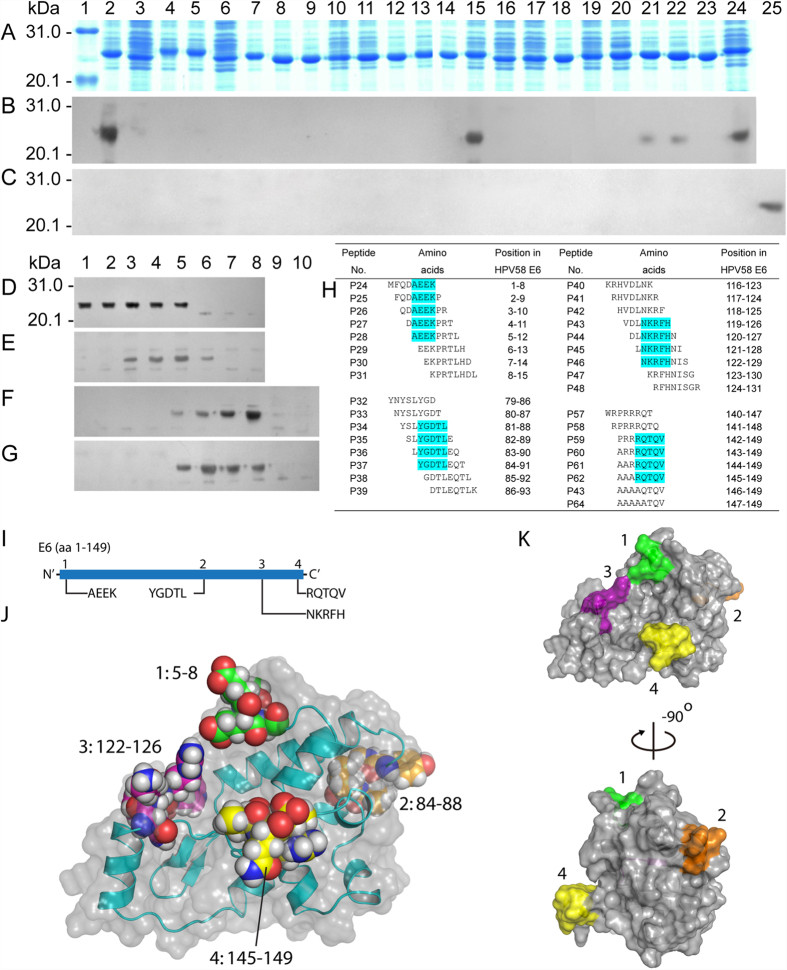
Epitome mapping of HPV58-E6. (**A**) SDS-PAGE analysis of *E. coli-*expressed r-15mer peptides fused with GST188. Lane 1, prestained protein markers; lanes 2–24, P1–P23 recombinant fusion proteins encompassing overlapping peptides. (**B**) Western blotting of P1–P23 using immune serum sample from rabbit (No. 2) immunized with *E. coli*-expressed r-E6. (**C**) Western blotting using preimmune serum as negative control. Lane 25, positive control using reactive P13 of E7 (Fig. 2B). (**D–G**) Western blotting of overlapping 8mer peptides. (**H**) The table analyzing each minimum epitope motif shown by cyan. (**I**) Linear schematic diagram of 4 BCEs on HPV58-E6 protein. (**J,K**) Positioning of mapped BCEs (Nos 1–4) on the predicted 3D structures of E6, which are highlighted with various colors (1, green; 2, orange; 3, purple; and 4, yellow) in J. The 3D structure prediction of E6 is according to the method in ref. 18, as well as J and K are generated using molecular graphics system PyMOL. The cropped blots were run under the same experimental conditions and the full length blots for the pictures shown above can be viewed in Supplementary Fig. S3.

Western blot studies with rabbit pAbs against r-E6 (rabbit number 2) revealed five reactive 15/17mer peptides corresponding to E6 in the first round of antigenic peptide mapping (Fig. 1A–C). These peptides also reacted with the pAbs generated against r-E6 in 3 additional rabbits except P14. Further, using overlapping 8mer r-peptides, four precise BCEs (named E6-1, E6-2, E6-3, and E6-4) were identified for reactive P1, P14, P20/P21, and P23 according to their common sequences within reactive 8mer peptides (Fig. 1D–H) in the second round of fine epitope characterization. Of them, E6–3 motif was located in the overlapping region of P20 and P21. To determine E6-4 minimal motif of a reactive 8 mer peptide (P59) observed during fine motif mapping of P23, five extra 8mer peptides (P60–P64) were employed, wherein the respective aa residue from N terminal of P59 was sequentially substituted with alanine (A).

The fine epitome map of HPV58-E6 (Fig. 1I) showed two obvious features: i) the distribution pattern of 4 BCEs presenting disperse state, and ii) except for one epitope at the N-terminal of E6, rest being situated in the second half of the protein. These features were similar with those of four antigenic peptides on HPV18-E6 as delineated by using pAbs raised in rabbit^8^. It is also often one of the major aims to determine antibody accessible BCEs on the target protein or surface of the virus following epitope-mapping using viral-infected animals antisera^19^,^20^. Thus, we investigated antibody accessibility of each mapped fine BCE through analysis of structural bioinformatics due to the weak immunogenicity of HPV compared with other viruses^21^, which is commonly used presently for identification of the antibody epitope^19^,^22^,^23^. These results suggest that 4 BCEs on E6 protein are located out of longer α-helical domains on predicted three-dimensional (3D) structure or close to one end of them (Fig. 1J) and they are exposed on the surface of the predicted 3D structure (Fig. 1K).

### E7-epitome

**Figure 2:**
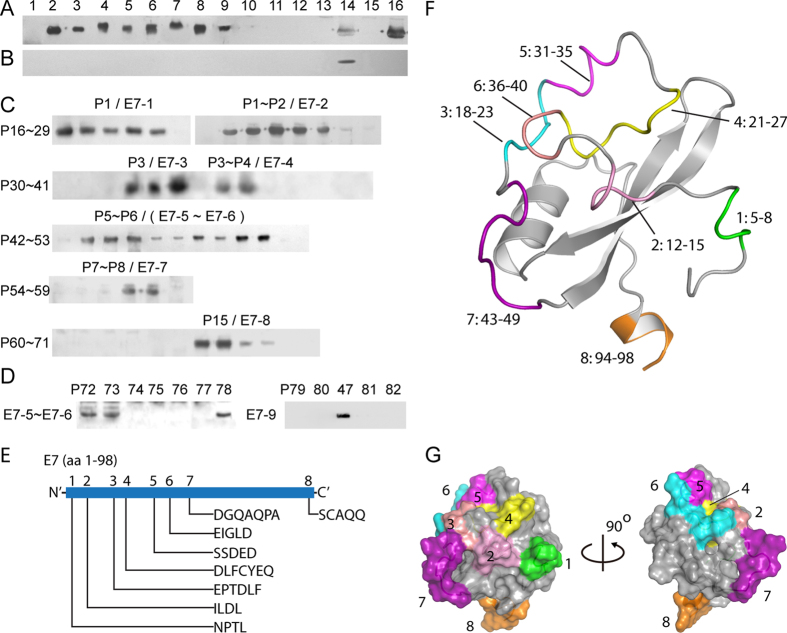
Epitome mapping of HPV58-E7. (**A**) Western blotting of 15mer peptides using immune serum samples from rabbit (No. 1) immunized with *E. coli*-expressed r-E7. Lane 1, pre-stained protein markers; lanes 2–16, P1–P15. (**B**) Preimmune serum control. Lane 14, showing reactivity with P13. (**C**) Western blotting of 5 sets of overlapping 8mer peptides (P16–P29, P30–P41, P42–P53, P54–P59 and P60–P71); common amino acid sequence analysis within reactive peptides is shown in Supplementary Table S1. (**D**) Western blotting of 2 sets of overlapping 6/8mer peptides (P72–P78 for E7-5 and E7-6, and P79–P82 for E7-9). (**E**) Linear schematic diagram of 8 BCEs on HPV58-E7 protein. (**F,G**) Positioning of mapped BCEs (Nos 1–8) on the predicted 3D structures, which all are highlighted with various colors (1, green; 2, pink; 3, salmon; 4, yellow; 5, magenta; 6, cyan; 7, purple and 8, orange) in F and G. The 3D structure prediction of E7 is according to the protocol in ref. 25, as well as F and G are generated using molecular graphics system PyMOL. The cropped blots were run under the same experimental conditions and the full length blots for the pictures shown above can be viewed in Supplementary Fig. S4.

E7 is another key early oncoprotein of HR-HPV and useful for determining HPV seroprevalence^4^,^8^. Using rabbit E7-pAbs (Supplementary Figures S1B and S2B), the E7-epitome was revealed following two rounds of epitope mapping, which consisted of 8 BCEs (named E7-1 to E7-8) mapped from 9 reactive 15mer peptides (Fig. 2A–C, and Supplementary Table S1 for all used 6/8mer peptide sequences, P16–P78), but not including a BCE (E7-9) uncounted in E7-epitome as it is telescoped within the sequences of E7-5 and E7-6. However, the process of each fine BCE mapping was more complicated than E6-epitome mapping due to the existence of continuous 8 reactive 15mer (P1-P8) during antigenic peptide mapping and 9 reactive 8mer peptides (P43–P51) in fine epitope identification.

For example, to determine how many BCEs were present in 8 reactive overlapping 15mer peptides (Fig. 2A), more overlapping 8mer peptides of adjacent reactive peptides were expressed after fine epitope mapping of P1, P3, P5, and P7 (if a mapped BCE motif was not located in the overlapping region between neighboring peptides); to distinguish borderline of two BCEs (E7-5 and E7-6) existing in 9 reactive octapeptides (P42–53, Fig. 2C; Supplementary Table S1), 7 shorter peptides (P72–P78 for E7-5 and E7-6) were expressed as possible epitope motifs based on the fact that one linear BCE consists of minimum 3 or maximum 8 aa residues^15^,^24^. As a result of the latter, the borderlines of mapping E7-5 and E7-6 were determined as P43–P46 and P48–P51, and the motifs of E7-5 and E7-6 were finally identified as pentapeptides SSDED and EIGLD, also including further finding another 8mer-BCE (E7-9, SDEDEIGL) of reactive P47 using 4 shorter peptides of P79–P82 (Fig. 2D and Supplementary Table S1).

The epitome of E7 protein with 98 aa in length showed an individual distribution of mapped BCEs too, but contrary to E6-epitome, the majority (7/8) of mapped BCEs are clustered at the N-terminal part of E7 (Fig. 2E). This observation is similar with mapping results of HPV16 and 18-E7 using respective monoclonal antibodies (mAbs), although the latter failed to show definite BCE number^5^,^6^. As shown in Fig. 2F predicted according to the protocol of MODELLER^25^, no BCE exists at the regions of predicted two β- sheets (no significant α-helix in the predicted structure) in the 3D model of E7, and all eight BCEs are located onto surface of the protein in their 3D positioning analysis, suggesting that these may be antibody-accessible BCEs (Fig. 2G).

### L1-epitome

**Figure 3:**
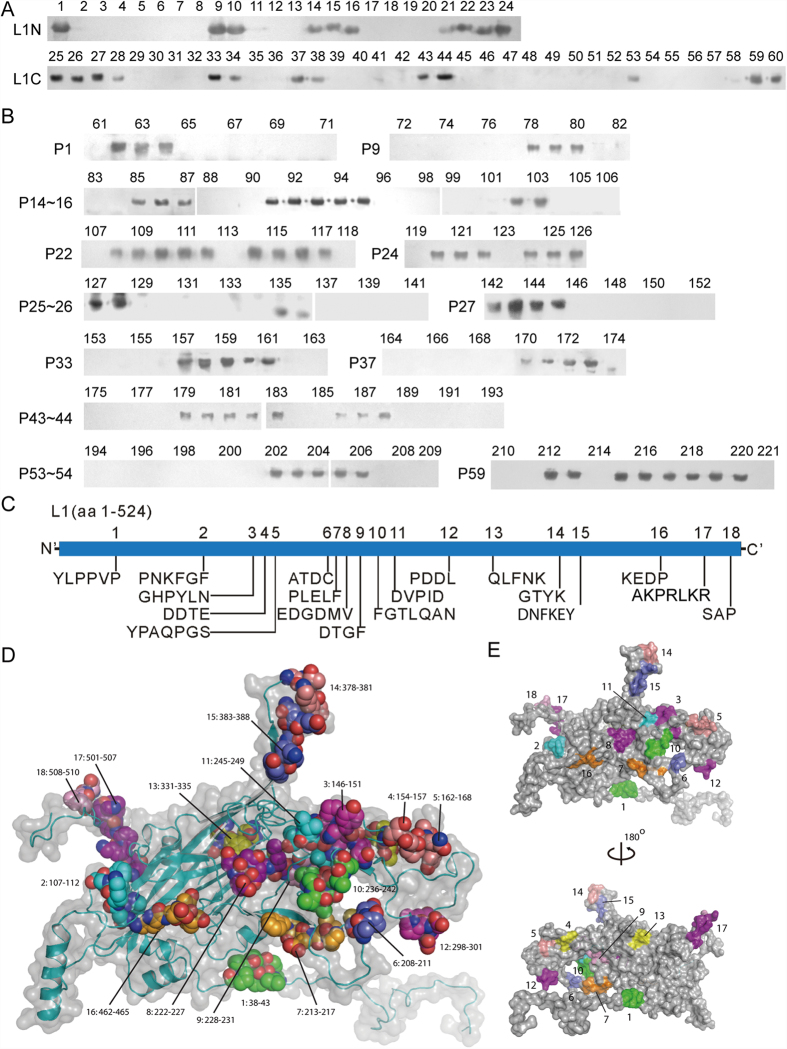
Epitome mapping of HPV58-L1. (**A**) Western blotting of overlapping 18mer peptides using immune serum samples from rabbits (Nos 2 and 3) immunized with *E. coli*-expressed r-L1N or r-L1C. P1-P24, reactivity of recombinant peptides with pAbs against L1N; P25–P60, with pAbs against L1C (negative control results of P1–P60 not shown). (**B**) Western blotting of 12 sets of overlapping 8mer peptides corresponding to immunoreactive 18mer peptides. P61–P221, 8mer peptides were used in identifying 18 BCEs. All 8mer peptides sequences corresponding to reactive 18mer peptides and analysis of the common sequence within reactive 8mer peptides are shown in Supplementary Table S2. (**C**) Linear schematic diagram of mapped 18 BCEs on L1 protein. (**D,E**) The positioning of the mapped 18 BCEs (Nos 1–18) on the predicted 3D structure of L1 protein. All BCEs exposed on the surface of the 3D structure are highlighted with various colors (1/10, green; 2/11, cyan; 3/12, magenta; 4/13, yellow; 5/14, salmon; 6/15, slate; 7/16, orange; 8/17, purple and 9/18, pink) in E. The 3D structure prediction of L1 is according to the method in ref. 27, as well as D and E are generated using molecular graphics system PyMOL. The cropped blots were run under the same experimental conditions and the full length blots for the pictures shown above can be viewed in Supplementary Fig. S5.

Major capsid L1 protein is the most important target antigen for the development of prophylactic HPV vaccines^26^ and serological diagnostic reagents. Up to now, only limited antigenic peptides on L1 protein of HPVs were reported utilizing sera of patients with HPV16-carrying cervical cancer, and pAbs or mAbs raised against L1-VLPs^3^,^7^,^10^,^11^,^12^,^13^. The HPV58-L1 epitome consisting of 18 BCEs (named L1-1–L1-18) was decoded after two rounds of epitope mapping by using rabbit pAbs against *E. coli*-expressed r-L1N (aa 36–234) and r-L1C (aa 227–524) (Supplementary Figures S1C,D and S2C,D) as well as 221 overlapping 18mer/8mer peptides (P1–P221, Fig. 3A–C and Supplementary Table S2), not including potential BCEs that may be present in the N-terminal peptide (aa 1–35). The positioning of mapped 18 L1-BCEs on 3D structure has been shown in Fig. 3D,E, wherein 3D structure is predicted according to the method in Rosetta3^27^. All mapped BCEs were mainly located at the loop region of 3D model (Fig. 3D) and exposed on the surface of predicted 3D structure (Fig. 3E), suggesting that they all may be antibody-accessible BCEs.

Meanwhile, the statistical analysis may allow identifying immunodominant epitopes in decoded L1-epitome that will be capable of inducing antibodies in all the immunized rabbits. The L1-5 to L1-13 epitopes gathered in central region of the molecule (Fig. 3C) may be preferential candidates when designing either multi-epitope peptide vaccine of HPV58-L1 or single synthetic BCE peptide/multi-epitope r-peptide antigen for diagnostic use in ELISA and/or peptides based chip test^28^.

### Reactivity profile of the mapped BCEs with pAbs from different immunized rabbits

The reliability of revealed IgG-epitome depends upon the preparation of rabbit/mouse antisera, because sometime the antibody repertoire against a given protein may vary in different individuals of the same species. All mapped BCEs of E6 and E7 except for BCEs E6-2 and E7-8 generated antibodies in two out of the 4 and 3 rabbits immunized with r-E6 and r-E7 respectively (Table 1), suggesting the consistency of immune response generated in the immunized rabbits. However, several marked discrepancies of the responses to L1N and L1C were found in each immunized group, e.g. BCEs L1-3, L1-4, and L1-14 to L1-18 elicited their respective antibodies only in one (Nos 8 and 12) of 3 rabbits, but L1-5 to L1-13 did in all immunized rabbits, of which L1-9 generated antibodies only in 5 out of the 6 immunized rabbits (Table 1). It suggests that one need to immunize at least three rabbits for revealing whole epitome of a target protein so as to avoid losing one or more BCEs.

### Conservativeness and specificity of mapped BCEs

It is extremely important to determine antibody specificity and conservativeness of each mapped epitope among viral homologous proteins^10^,^11^,^12^ as the former would be critical parameter for diagnostic tools used in ELISA, chip test and/or Western blotting for diagnosing antibodies to type-specific viral infection. The latter could be used as potential candidates for developing ‘universal’ prophylactic peptide vaccine^22^,^23^,^29^. Although this goal can be achieved by using many approaches, such as alignment of mapped antigenic peptide sequence with conservative domain of homologues, and/or testing their cross-reactivity with other homologous synthetic peptides, r-protein, and/or mimicking natural protein such as L1-VLPs^11^,^12^. Determination of such epitopes, including their antibody-neutralizing ability, are likely to be few as the data may be incomplete or insufficient in most cases owing to the limitation of tested HPV types numbers and *in vivo* and *in vitro* experimental conditions^10^,^11^,^12^,^13^,^30^.

**Figure 4:**
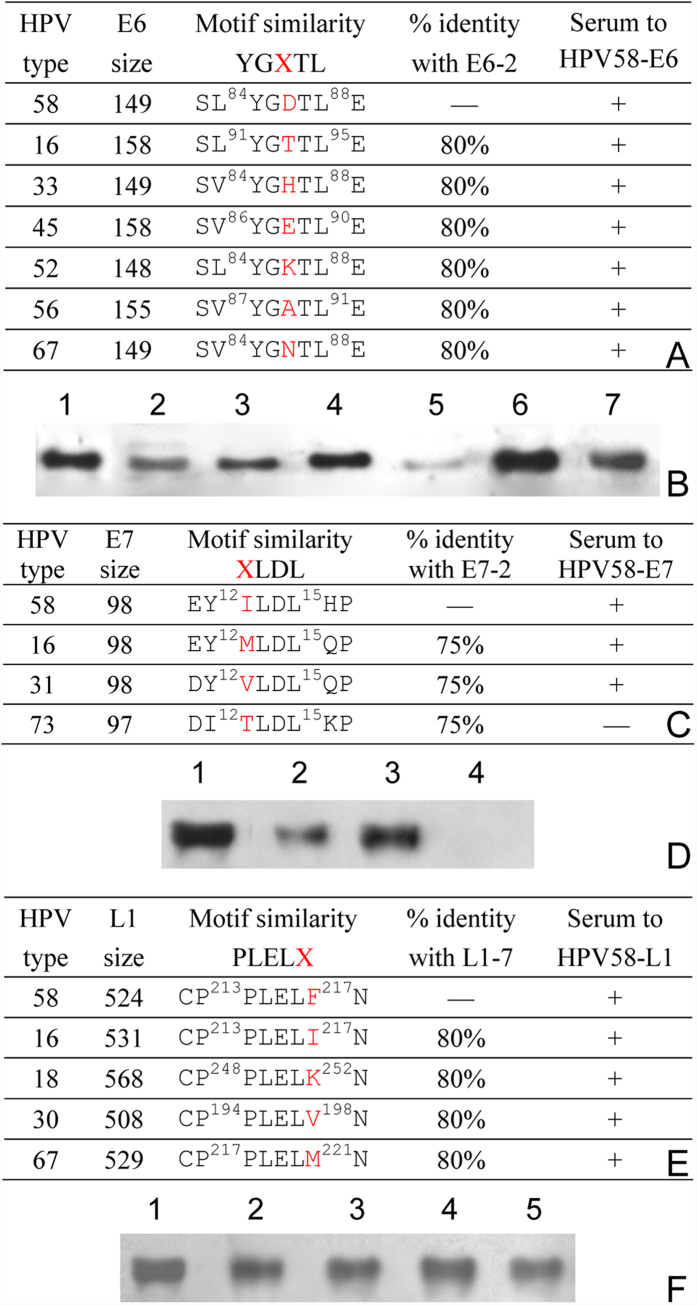
Cross-reactivity of three conserved BCE antibodies of HPV58 to various HPVs. (**A,C,E**) Sequence alignments of E6-2, E7-2 and L1-7 with their homologous proteins. (**B,D,F**) Western blots of each BCE antibody with representative octapeptides having one residue mutation at the X site, respectively. The cropped blots were run under the same experimental conditions and the full length blots for the pictures shown above can be viewed in Supplementary Fig. S6.

Now, it has become very easy, rapid and sufficiently effective to realize such goals through aa sequence alignment of homologous proteins and identification of each antibody-recognizing minimal motif^19^,^20^,^31^. Using the convenient method, thirteen of the total 30 BCEs in mapped three epitomes are found to be 100% conserved, and three BCEs are highly conserved (being a residue mutation at the X position of YGD/XTL for E6-2, I/XLDL for E7-2 and PLELF/X for L1-7 motifs) among various known and probable HR-HPVs [Table 2 and Supplementary Table S3 that mainly includes low risk (LR-) HPVs and risk-unknown HPV types], of which three (L1-4, L1-7, and L1-13) are broadly antibody cross-reactive BCEs that cover most HR-HPVs, remaining 14 BCEs are type-specific for HPV58. Three highly conserved epitopes were confirmed by Western blotting to be cross-reactive along with representative similar peptides from other HR-HPVs using each corresponding antisera (Fig. 4), of which only a conserved TLDL peptide of HPV73 did not react (Fig. 4D). It may be of interest to note that broadly antibody cross-reactive BCE, L1-7 (PLELF) is also present in HR-HPVs such as HPV16 (PLELI) and HPV18 (PLELK), and also react with the antibody in Western blot (Fig. 4E,F). Further E6-2 (YGDTL) and E7-2 (ILDL) discovered from HPV58 are also present in HPV16 (Fig. 4A–D). However, other two similar peptides corresponding to E7-1 (N/XPTL) and L1-15 (D/XNFKEY) in some HPVs were not recognized (data not shown). These observations suggest that although several mapped BCE motifs may be highly conserved among other HPVs, they are not always antibody cross-reactive epitopes.

Overall, these analytical and experimental results indicate the importance of conducting fine epitope motif identification during epitope mapping, which not only can help in revealing whole IgG-epitome of a target antigen, but also will facilitate to decipher the conservativeness and/or specificity of each mapped BCE among its homologous proteins and to find universal BCEs in particular.

### Reactivity of mapped L1-BCEs with murine antisera to HPV58 L1-VLPs

**Figure 5:**
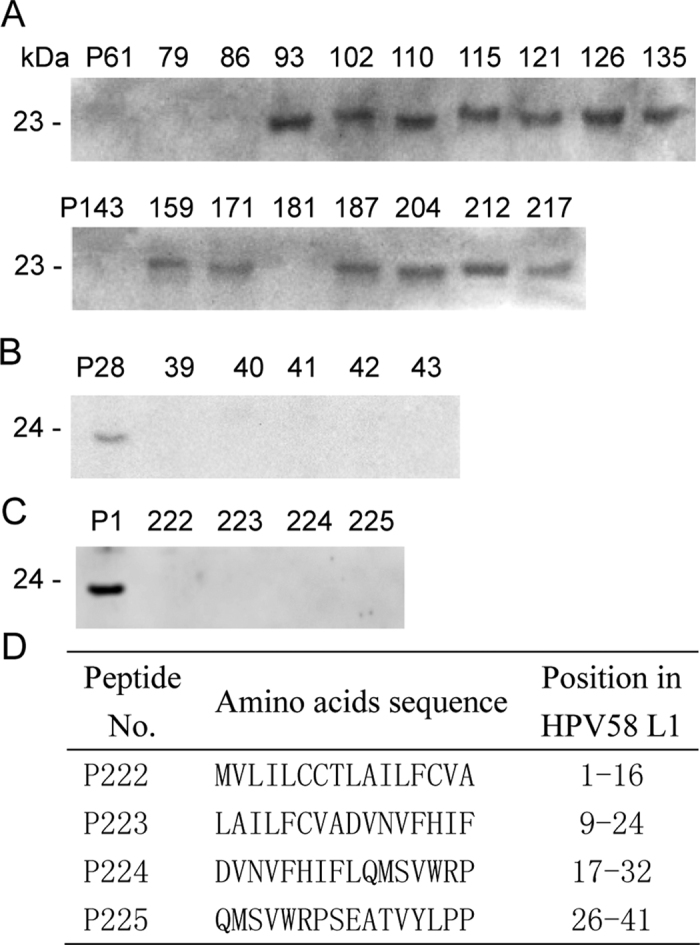
The reactivity of mapped 18 BCEs with murine antisera to HPV58 L1-VLPs. **(A)** Western blotting of rabbit-recognized 18 BCE peptides using murine antisera to HPV58 L1-VLPs. **(B)** Western blotting of five nonreactive 18mer peptides to rabbit L1C-antisera using murine antisera to HPV58 L1-VLPs. **(C)** Epitope mapping of the N-terminal region (aa 1-41) of L1 antigen using 30 μL of murine antisera to HPV58 L1-VLPs that contain 2 μL of rabbit (No. 8) antiserum to r-L1N. P1 was the positive control. **(D)** Four overlapping peptide sequences used in Fig. 5C. The cropped blots were run under the same experimental conditions and the full length blots for the pictures shown above can be viewed in Supplementary Fig. S7.

Because surface-exposed BCEs of virus/VLPs will induce antibodies against respective BCEs in the infected/inoculated animals, the pooled mice antiserum raised against HPV58 L1-VLPs^32^ was employed to further confirm the result of L1-epitome decoding by using 18 of the reactive 8mer peptides pertaining to each BCE motif. Western blot test showed that 13 of mapped 18 BCEs could be recognized by pooled mice antiserum (Fig. 5A) and they include 5 of 7 BCEs to induce antibodies in single rabbit (Nos 10 or 13), suggesting better consistency of immune responses to denatured r-L1 protein and similar natural antigen of L1 between rabbit and mouse immune systems. More importantly, these reactive BCEs contain almost all deduced immunodominant BCEs (L1-5 to L1-13) and several ‘promiscuous’ BCEs (L1-4, L1-7, and L1-13) covering most known and probable HR-HPVs (Table 2 and Supplementary S3), which may be used as candidates when designing universal preventive peptide vaccine. Moreover, the Western blot test using murine antisera supports the 3D positioning result of mapped L1 BCEs, when at least 13 BCEs of them can be definitely located on the surface of L1 protein, although the remaining five non-reactive BCEs remain to be confirmed using rabbit antiserum to HPV58 L1-VLPs, if it could be available.

Except for above comparability, there were some differences to recognize BCEs on capsid L1 protein between the two species. For instance, murine antisera did not react to the other five 8mer peptides (P61, 79, 86, 143, and 181 in Fig. 5A), also including their corresponding 18mer peptides P1, 9, 10, 14, 27, and 43 (data not shown), but could react to the P28 in selected five nonreactive 18mer peptides to rabbit L1N/L1C-antisera (Fig. 5B), indicating that recognizing antigenic sites or BCE motifs on same antigen are sometimes different. These observations may be able to explain why murine antisera reacted to most of BCEs within rabbit IgG-epitome, but not all. Additionally, it was observed that there was no BCE on the N-terminal region (aa 1-41) of L1 antigen (Fig. 5C) when using murine antisera and four overlapping peptides (P222–P225; Fig. 5D).

### E6-2 pentapeptide marker for HR-HPVs

To date, over 150 HPV genotypes have been described, of which about 40 can infect human genital tract mucosa and at least 12 were determined as HR-HPV types associated frequently with cervical cancer^18^,^33^,^34^. Interestingly, a highly conserved pentapeptide (YGD/XTL) of E6-2 motif exists in the most common HR-HPV types 16, 18, 31, 33, 35, 45, 52, and 58 with a prevalence of 91% worldwide^33^,^34^ in the multiple sequence alignment at epitope level (Table 2 and Fig. 4A). Even within 12 HR-HPVs defined by the World Health Organization (WHO)^18^, HR-HPV39 is the only exception.

To further establish whether it is a common characteristic of HR-HPVs, the E6 protein of known representative LR-HPVs^32^,^35^,^36^ were also used to conduct their sequence analysis. As shown in Supplementary Table S3, which includes several probable HR-HPVs such as HPV67 and 69 etc, the generally accepted LR-HPV types 6, 11, 40, 42, 43, and 44 do not have conservative E6-2 sequence. Additionally, owing to high variations of HPV, it was further analyzed whether there are aa mutations related with E6-2 motif for available HR-HPV variants before it is recommended as a reference standard for determining HR-HPV type(s). As a result, the D/E mutation exists within thirteen of 130 HPV58 variants^37^,^38^,^39^,^40^,^41^ and three of 7 HPV33^37^ variants that only happened at the X site of E6-2 motif (Table 3), but there is no aa mutation associated with YGD/XTL pentapeptide sequence in all HR-HPV type 16, 18, and 52 variants (Supplementary Table S4).

Based on these findings, the conservative YGD/XTL pentapeptide should be a property of 11 determined HR-HPVs contained within species 5, 7, and 9 of the *Alpha-papilloma viruses* at their phylogenetic tree^42^, although its biological function is not clear at present. Further, it raises the possibility to use the pentapeptide as the first biomarker of HR-HPVs on the classification basis of epidemiologic studies and phylogenetic analysis. Obviously, it will aid in classifying those HR- or LR-HPVs undetermined for long time to use it as reference standard. For example, of probable or undetermined HPV types, 26, 34, 66, 67, 69, 73, 82, 85, and 97 can be definitely classified as HR-HPVs according to its existence within them (Table 2 and Supplementary Table S3).

Interestingly, according to the SVYATTLE sequence of known HR-HPV39 (Fig. 4A) within species 7, if the E6-2 motif is further extended to octapeptide (SVYXXTLE and SVYGXXLE) as the second and third biomarkers, those probable HR-HPV53 (SVYGASLE), 68 (SVYATTLE), and 70 (SVYATTLE) present in species 6 and 7 are also regarded as HR-HPVs, because such two peptide sequences are not present in all other cloned HPVs. In contrast, HPV types 30, 54, 57, 61, 62, 72, 74, 81, 89, 91, etc could be considered as LR-HPV types according to two reference criteria. In short, the application of above one or more penta/octa-peptide biomarkers in HR- and LR-HPVs classification will contribute to ascertain those currently unknown HPVs should be classified as HR- or LR-types in clinical diagnosis and epidemiological screening of HPV infection and cervical cancer, and to improve patient care and novel vaccine design strategies in future.

## Discussion

Although the development of synthetic peptides vaccines based on few BCEs has a long history, there has been no successful precedent so far due to insufficient protective effect for humans use. Recently, there appeared the trend of constructing multi-epitope vaccinogen^43^,^44^,^45^,^46^, but such studies still have same problem to incorporate more selectable BCEs from a given antigen. Therefore, it is crucial to reveal all BCEs on a target antigen for the development of really effective multivalent peptide vaccine in future, but this goal was not achieved before establishing the improved method of biosynthetic peptide^14^,^15^. The peptide biosynthetic approach described herein to mapped linear BCEs has several advantages that includes i) it is relatively simple as it employ thermo-inducible expression system to express peptides fused with truncated GST as carrier; cell lysate can be used directly to perform Western blot without the necessity to purify recombinant peptides, ii) it is relatively cheaper as compared to making synthetic peptides, iii) it is reliable as the blotted bands of the peptides fused with truncated GST are located within antigenic weak area of bacterial proteins, and iv) easy adaptability for general laboratories. One of the possible disadvantages is to sequence each of the recombinant clones to ensure correct sequence. In short, the improved peptide biosynthetic method specific for epitope mapping is simple, cost-effective and reliable compared with other methods such as those using overlapping synthetic peptides or peptide expression libraries in micro-organisms. In the present study, the epitome mapping results proved the feasibility and creditability of decoding a whole IgG-epitome for an antigen, which may facilitate the development of peptides based vaccine.

Earlier studies on M2 matrix protein being highly conserved among influenza A viruses initially suggested possibility to develop ‘universal’ prophylactic flu vaccine^47^,^48^. Now current favored strategy is to search broadly cross-neutralizing mAbs and then to map their BCEs^22^,^23^,^29^. It should also be a goal to develop universal vaccine covering most or all HR-HPVs. The ongoing development of 9-valent HPV vaccine combined nine L1-VLPs from 7 HR- and 2 LR-HPVs that included HR-HPV58 L1-VLPs^49^. Clearly, to reduce vaccine cost and extend range of preventing HR-HPV types, constructing universal multivalent HPV vaccine may be a potential alternative approach due to the existence of cross-neutralizing BCEs on HPV L1 and L2 proteins^11^,^12^,^13^,^30^, though limited information is available regarding the cross-reactive BCEs and among HR-HPVs^11^,^30^ that are covered at present.

Besides type-specific BCEs, this study shows the possibility to find more conserved BCEs capable of covering most or nearly all HR-HPVs via conservative analysis of each BCE after epitome mapping. For major capsid L1 protein, despite not finding a conservative BCE to cover all HR-HPVs due to about 10% difference of L1 gene among each HPV type^42^, more valuable information is available from Table 2 and Supplementary Table S3. For example, three of the analyzed 11 conserved BCEs exist within most HR-HPVs including HPV16, 18, 45, and 52 that are prevalent worldwide^33^ or in China and Asia^50^, even within two typical LR-HPVs (HPV6 and 11), suggesting that they are potential candidates for designing universal multi-epitope vaccine against major or entire HR-HPVs. Of course, achieving such a goal still requires finding additional conservative BCEs through L1-epitomics study of more HR-HPVs such as HR-HPV16/18/45/52, etc.

There is evidence to demonstrate that: i) VLPs can elicit high-titer neutralizing antibodies in animal modle^32^,^51^; ii) though linear BCEs was usually considered to be mostly nonneutralizing^52^, it has been found that one or more linear neutralizing epitopes are present in capsid proteins of various HPVs^11^,^12^,^13^,^30^, of which two come from HPV16^11^; and iii) antibodies induced by Gardasil and Cervarix HPV VLPs vaccines have been proved to be effective for preventing HPVs infection^26^,^49^, for which the protective validity should include the contributions of many nonconformational neutralizing antibodies from HPV16/18 or HPV6/11/16/18-L1. Thus, based on these observations, at least 13 surface-exposed BCEs including 3 broadly antibody cross-reactive BCEs should contain one or more potential antibody-protecting BCEs within HPV58 L1-epitome. Interestingly, 3 broadly antibody cross-reactive BCEs mapped on HPV58 L1 as described herein are also present in HPV16/18. Clearly, it is imperative to further identify antibody-neutralizing BCEs before they are considered to be used as candidates when designing HPV multi-epitope vaccine.

The mucosal epithelia-infecting HPVs are presently divided into two categories of HR and LR types. It is based mainly on epidemiologic classification and phylogenetic grouping, which are linked with cervical cancer and/or HPV natural history^33^,^42^, and also including some evidence and indicators of biological functions or features for HR-HPV E5, E6 and E7 relevant to carcinogenicity^53^. However, except 12 HR-HPV types (16, 18, 31, 33, 35, 39, 45, 51, 52, 56, 58, and 59) accepted generally^18^, the current data on oncogenicity of many HPV types is ambiguous. For example, the classification of HPV26, 54, 67, 69, 70, 82, etc in literature and in clinical diagnosis were completely different^34^,^35^,^54^. In short, due to rare prevalence or limited and inconsistent carcinogenic evidence in humans the “HR” or “LR” status of HPVs such as 26, 30, 34, 53, 54, 62, 66–70, 73, 85, 97, etc could not yet be determined^18^,^33^,^34^. Obviously, the findings of conserved pentapeptide YGD/XTL only present in known HR-HPVs, including other related two octapeptide markers, have important implication for helping to delineate the oncogenic risks of those probable and unknown HR- or LR-HPV types.

Previous studies showed that most of the HPV infections clear spontaneously without sequelae in around 1 to 2 years through T-cell mediated immune responses to early oncogenic E6 and E7^55^,^56^. Due to lack of specific and sensitive methods to test E6/E7 antibodies, it is not clear whether humoral immune response to E6 and/or E7 is also associated with the self-clearance. Our first mapped fine epitomes of HPV58-E6 and E7 not only improve our understanding of the antigenic peculiarity on two carcinogenic proteins, but will facilitate in designing and developing specific and sensitive reagents or peptide chip^28^ leading to better understanding of the natural clearance of HPV58 infection and its carcinogenicity. The epitomes of HPV16, 18, and 45-E7 has been also decoded by using rabbit pAbs to r-E7 in another ongoing work, which revealed 7/6/4 BCEs on each E7 and their quite similar distribution profile as observed in HPV58-E7 (Fig. 2E), respectively (unpublished data). These consistent results, together with previous related studies^5^,^6^, suggest that the feature of HPV58-E7 epitome are common among four of HR-HPVs including HPV16/18 and thus, might be useful as a valuable parameter to evaluate E7 carcinogenic risk, although not clear whether it is associated with self-clearance following HPV infection.

In conclusion, we identified all linear BCEs on three target proteins from HPV58 and revealed their specificity, conservativeness and antibody accessibility; especially found three universal BCEs in L1-epitome, one E6-2 biomarker of HR-HPVs and unique contribution of mapped BCEs on E7. These results not only will facilitate the development of multi-epitope diagnostic reagents and universal preventive HPV peptide vaccine based on broadly crossreactive BCEs of L1, but also display the feasibility and significance of epitome decoding for a viral-encoded several key proteins.

## Materials and Methods

### Plasmids and antibodies

The plasmids, pBV221 (VT1884) and pRSET-A expressing His_6_-tag (V35220) were purchased from YouBio (Hunan, China) and Life Tech (Shanghai, China). The plasmids, pXXGST-1 and pXXGST-2 were constructed as described earlier^14^,^15^. The pLink322-HPV58 plasmid^57^ was obtained from Dr. Toshihik Matsukura (National Institute of Infectious Diseases, Department of Dermatology, Nippon Medical School, Tokyo, Japan). The monoclonal antibody (mAb) against His_6_-tag (ab77824) was purchased from Abcan (Shanghai, China). The murine antisera against HPV58 L1-VLPs^32^ were provided by Prof. J. Lou (Research and Development Department, Shanghai Institute of Biological Products, Shanghai 200052, China).

### Expression of E6, E7, L1N [amino acid (aa) 36-234] and L1C (aa 227-524)

The thermo-inducible pBV221 plasmid was used to express E6, E7 and two fragments of L1 in prokaryotic system. Briefly, various steps involved in their expression were as follows: i) the DNA fragment with *BamH* I and TAA-*Sal* I cohesive end on their 5′ and 3′ ends encoding respective protein was PCR-amplified from pLink322-HPV58 using a pair of designed primers; ii) the sequenced-DNA fragment was inserted into the *BamH* I and *Sal* I sites downstream of the pBV221; iii) the resultant recombinant plasmid expressing respective r-protein was transformed into the BL21[DE3]pLysS *E. coli* strain (Novagen Inc., Madison, USA); iv) several recombinant clones were picked-up from Luria Broth (LB) plate and first grown in 3 mL of LB containing 100 μg mL^−1^ ampicillin at 30 °C with continuous shaking at 200 rpm overnight. Next day, 60 μL of cell suspension was inoculated in 3 mL of fresh LB and grown until the cell density reached 0.6–0.8 at OD_600_, and then further grown for 4 h at 42 °C to induce expression of r-protein; v) the cell pellets harvested from induced clones were first used to run SDS-PAGE gel for screening positive recombinant clone: the uninduced cell total protein was used as negative control (Supplementary Fig. S1A–D); and vi) finally the clone expressing r-protein was again sequenced. The isopropyl-β-D-thiogalactopyranoside (IPTG)-inducing pRSET-A plasmid was used to express r-protein with His_6_-tag, which utilized basically same steps as described above, but its expression was further confirmed by employing the mAb against His_6_-tag (Supplementary Fig. S1E).

### Biosynthesis of 8mer and 15/18mer peptides

Using GST188 as carrier, three sets of overlapping 15/17/18mer peptides with an overlap of 9/10 aa residues for E6, E7 and L1 proteins, and series of overlapping 8mer peptides with 7 aa residues overlap for each reactive 15/17/18mer peptides (all sequences in Fig. 1H; Supplementary Tables S1 and S2) identified in epitome mapping, were expressed as fusion proteins as described earlier^14^,^15^. Briefly, the synthesized annealed DNA fragments encoding each short peptide, incorporating *BamH* I and TAA-*Sal* I cohesive end on their 5′ and 3′ ends, were inserted into the *BamH* I and *Sal* I sites downstream of GST188 gene in pXXGST-1 plasmid. BL21(DE3)(pLysS) *E. coli* cells were transformed with recombinant plasmids, positive clones were picked-up to express fusion peptides and harvested total cell proteins of the induced clone according to steps (ii to v) described above. Each harvested cell pellet was first used to run 15% SDS-PAGE along with the pellet containing GST188 protein expressed by pXXGST-2 plasmid, and then determined each positive recombinant clone according to electrophoretic mobility of each fusion protein and GST188, since there is 1 kDa difference between their molecular mass even when expressing 8mer peptide fusion protein. Finally, the positive clones expressing 8/15/17/18mer peptides as fusion protein were confirmed by sequencing of inserted encoding DNA fragments. All cell pellets thus obtained containing each fusion peptide were stored at −20 °C.

### Purification of expressed r-E6, -E7, -L1N and -L1C proteins

The expressed recombinant proteins were harvested using the method of PAGE-preparation^58^. The steps to purify recombinant proteins involved: i) cell pellet (2 g) obtained from induced culture of the respective clone was suspended in 5 mL of sonication buffer (50 mM Tris–HCl pH 8.0, 1 mM EDTA, 100 mM NaCl and 1 mM PMSF) followed by sonication at 25 kHz, 400 W for 140 cycles (duration 3 sec with an interval of 3 sec every time) at 4 °C; ii) The inclusion bodies were recovered by centrifugation at 17,000 × g for 20 min at 4 °C, washed once with 50 mL of sonication buffer containing 0.5% Triton-X100 and 25 mL of 1 M urea; iii) the purified inclusion bodies were solubilized in 25 mL of 50 mM Tris–HCl buffer (pH 9.8) supplemented with 8 M urea and 70 mM β-mercaptoethanol; iv) the supernatant containing r-protein was finally collected after centrifugation at 17,400 × g for 20 min at 4 °C and stored at −70 °C prior to purification; vi) the supernatant was subjected to reversed electrophoresis by rearranging the gel-carrying plate when the bromophenol blue band reached 1–1.5 cm from the bottom of the gel, and 8 fractions of 3–4 mL each were collected after the bromophenol blue had run out of the gel into a collecting trough between the gel and a dialysis membrane used to isolate the upper tank buffer; vii) from each collected fraction, 0.2 mL was taken and the r-protein precipitated by sequentially adding trichloroacetic acid and acetone; viii) the sample was dissolved in 20 μL 4 x sample loading buffer (200 mM Tris-HCl, 8% SDS, 0.4% bromophenol blue, 40% glycerol and 400 mM β-mercaptoethanol), analyzed and quantified [together with 2 μg bovine serum albumin (BSA) standard purchased from Sigma-Aldrich Inc., St. Louis, IL, USA] by running SDS-PAGE; and ix) finally, the identified fractions were pooled and the precipitated r-protein stored at −70 °C prior to use as immunogen.

### Immunization of rabbits

All the experiments were approved by the Institutional Animal Care and Academic Committees for Shanghai Institute of Planned Parenthood Research, respectively. All the protocols were performed in accordance with the relevant guidelines and regulations. Fourteen male New Zealand white rabbits weighing 2.0 ± 0.5 kg were purchased from SIPPR-BK Lab Animal Ltd. Co. (Shanghai, China). Group of rabbits (n = 3/4) were immunized intramuscularly with 0.5 mg of purified respective r-proteins (0.5 mL) emulsified in 0.5 mL of complete Freund’s adjuvant (CFA) at multiple sites on the back of each animal. One rabbit used as control received only CFA. Three booster injections comprising of 0.25 mg r-protein per injection in incomplete Freund’s adjuvant (IFA) were administered at 3-week intervals. Animals were bled 7 days after each injection, serum separated and stored at −70 °C until use.

### ELISA

Serum antibody titers were determined by ELISA using respective r-protein as the antigen. ELISA plates (96-well; Sigma-Aldrich Inc.) were coated with 400 ng of r-E6, -E7, -L1N or -L1C protein/well, respectively. After overnight incubation at 4 °C, excess liquid was removed and non-specific sites were blocked by the addition of 200 μL of 3% BSA in PBS (pH 7.4) for 1 h at room temperature (RT). Following blocking, plates were washed three times in PBST (PBS supplemented with 0.05% Tween-20), and then serially diluted rabbit antisera in PBS were added and incubated for 2 h at RT. After washing the plates three times with PBST, goat anti-rabbit IgG conjugated to horseradish peroxidase (HRP) at an optimized dilution of 1:1000 in PBS was added to each well and incubated for 1 h at RT and washed as described above. Plates were incubated with 0.4 mg/mL orthophenylenediamine in 50 mM citrate phosphate buffer, pH 5.2 with 0.015% (v/v) H_2_O_2_ as the substrate to develop color. The reaction was stopped with 50 μL of 4 N H_2_SO_4_ and the absorbance read at 490 nm with 630 nm as reference filter using an ELX 800 Universal Micorplate Reader (Bio-TEK Instruments, Inc.).

### SDS-PAGE and Western blotting

Cell pellets obtained from 3 mL culture of expressed r-proteins and short peptide fusion proteins were boiled in 400 μL of 1x sample loading buffer (50 mM Tris-HCl, 2% SDS, 0.1% bromophenol blue, 10% glycerol and 100 mM β-mercaptoethanol) for 5 min and proteins were resolved by 15% SDS-PAGE under reducing conditions. Gels were either stained with Coomassie brilliant blue G-250 for analyzing the bands of fusion proteins or processed for Western blot by electrotransferring the proteins onto 0.2 μm nitrocellulose membrane (Whatman GmbH, Dossel, Germany). While epitome mapping, complete transfer of proteins was ensured by staining the nitrocellulose membrane with 0.1% (w/v) Ponceau S. Nitrocellulose membrane was subsequently processed for Western blotting using rabbit antisera against r-E6, -E7, -L1N, and -L1C as well as murine antisera against HPV58-L1 VLPs (1:300 dilution in PBS containing 0.05% Tween 20 and 1% skim milk powder) or preimmune rabbit/mouse serum samples. Specific antigen-antibody reaction on the membrane was visualized by using goat anti-rabbit IgG or goat anti-mouse IgG (only for identification of expressed r-E7) conjugated to HRP (Proteintech Group Inc., Chicago, IL, USA) at 1:2000-dilution. The blot was developed by using the enhanced chemiluminescence (ECL) plus Western blotting detection reagents (GE Healthcare, Buckinghamshire, UK) according to the manufacturer’s instructions.

### Sequence alignment of homologous HPVs

The aligned E6, E7 and L1 sequences of prototypes from HPV6 to HPV97 within Table 2 and Supplementary Table S3 are available from GenBank, NCBI. Their accession numbers are AF092932, M14119, K02718, X05015, X74472, X74474, J04353, M12732, NC_001587, M74117, M62849, X74478, M73236, AJ620205, U31788, X74479, M62877, X74481, X74482, U37488, EF177181, D90400, X77858, U31793, AY395706, U31794, D21208, DQ080079, AB027020, U21941, X94164, X94165, AJ620209, AB027021, AF131950, AF436128, AF419318, and DQ080080, respectively.

## Additional Information

**How to cite this article**: Xu, W.-X. *et al*. Epitomics: IgG-epitome decoding of E6, E7 and L1 proteins from oncogenic human papillomavirus type 58. *Sci. Rep.* **6**, 34686; doi: 10.1038/srep34686 (2016).

## Supplementary Material

Supplementary Information

## Figures and Tables

**Table 1:** Immune response comparison of mapped BCEs in rabbits of each immunized group.

**Immunogen**	**Rabbit No.**	**E6-1**	**E6-2**	**E6-3**	**E6-4**						
E6	1	+	−	+	+						
	2	+	+	+	+						
	3	+	+	+	+						
	4	+	−	+	+						
**Immunogen**	**Rabbit No.**	**E7-1**	**E7-2**	**E7-3**	**E7-4**	**E7-5**	**E7-6**	**E7-7**	**E7-8**	**E7-9**	
E7	5	+	+	+	+	+	+	+	−	+	
	6	+	+	+	+	+	+	+	+	+	
	7	+	+	+	+	+	+	+	+	+	
**Immunogen**	**Rabbit No.**	**L1-1**	**L1-2**	**L1-3**	**L1-4**	**L1-5**	**L1-6**	**L1-7**	**L1-8**	**L1-9**	
L1N	8	+	+	+	+	+	+	+	+	+	
	9	+	+	−	−	+	+	+	+	+	
	10	−	−	−	−	+	+	+	+	+	
**Immunogen**	**Rabbit No.**	**L1-9**	**L1-10**	**L1-11**	**L1-12**	**L1-13**	**L1-14**	**L1-15**	**L1-16**	**L1-17**	**L1-18**
L1C	11	−	+	+	+	+	−	−	−	−	−
	12	+	+	+	+	+	+	+	+	+	+
	13	+	+	+	+	+	−	−	−	−	−

“+” means each BCE can be recognized by the corresponding antiserum from respective immunized rabbit in Western blotting,

and “−” means negative response.

**Table 2:** Conservative analysis of HPV58 BCEs among known and probable HR-HPVs.

HPV58	HPV types and aligning sequence (aa range)																	
BCE No.	16	18	31	33	35	39	45	51	52	56	59	26**	53**	66**	68**	73**	82**	97**
E6-2*	91–95	86–90	84–88	84–88	84–88		86–90	84–88	84–88	87–91	86–90	84–88		87–97		85–89	84–88	86–90
E6-3				122–126					122–126									
E7-2*	12–15	12–15	12–15	12–15	12–15	12–15			12–15		12–15	12–15				12–15		12–15
E7-5				31–35								36–40						
E7-7				42–48														
L1-1	38–43		12–17	12–17					38–43									
L1-2			81–86	81–86	80–85				109–114							78–83		
L1-4	153–156	188–191	128–131	128–131	127–130	127–130	154–157	126–129	156–159	190–193	127–130	126–129	125–128	126–129		125–128	126–129	153–156
L1-7*	213–217	248–252	188–192	187–191	190–194	186–190	214–218	186–190			187–191	186–190	185–189		127–130	185–189	186–190	213–217
L1-8		257–262		196–201			223–228				196–201		194–199	195–200	187–191		195–200	222–227
L1-9			203–206	202–205	205–208			201–204	231–234	265–268		201–204	200–203	201–204			201–204	
L1-11				219–223					248–252			218–222				217–221		
L1-12	298–301			272–275													270–273	
L1-13		366–371		305–309		304–308	334–338		336–340	368–372	305–309	305–309	303–307	304–308	305–309	303–307		331–335
L1-16			438–441	436–439					467–470			438–441					438–441	
L1-18		548–550	487–489		433–435				526–518	549–551			490–492					513–515

An asterisk indicates those highly conserved cross-reactive epitopes of E6-2 (YGD/XTL), E7-2 (I/XLDL), and L1-7 (PLELF/X). Other BCEs were 100% conservative among some compared HPV homologous proteins. Two asterisks indicate probable HR-HPVs.

**Table 3:** Analysis of mutations of amino acids related with E6-2 in HPV58 and 33 variants.

HPV Types	Countries/ Regions	Refs	Mutation of amino acids and their position						D/E mutation/variant numbers
58	Japan	37	D86E^*^						1/1
	Hong Kong	38	E32Q	D86E^*^	K93N				8/32
	Taiwan	39	V46L	D86E^*^	K93N	N97D	R108K		2/9
	Liaoning, China	40	T11A	E25D	E32Q	V53L	D86E^*^	K93N	2/73
	Kunming, China	41	K93N						0/8
33	Japan	37	K8N	K35N	P36T	I73L	V83L	N86H^*^	3/7
			K93N	Q113R	A138V				

Asterisk indicates occurrence of D/E mutatilon.

## References

[b1] Walboomers J. M. . Human papillomavirus is a necessary cause of invasive cervical cancer worldwide. J Pathol. 189, 12–19 (1999). 10451482 10.1002/(SICI)1096-9896(199909)189:1<12::AID-PATH431>3.0.CO;2-F

[b2] Arbyn M. . Worldwide burden of cervical cancer in 2008. Ann Oncol. 22, 2675–2686 (2011). 21471563 10.1093/annonc/mdr015

[b3] Dillner J. . Mapping of linear epitopes of human papillomavirus type 16: the L1 and L2 open reading frames. J Cancer. 45, 529–535 (1990). 10.1002/ijc.2910450326 1689705

[b4] Dillner J. Mapping of linear epitopes of human papillomavirus type 16: the E1, E2, E4, E5, E6 and E7 open reading frames. J Cancer. 46, 703–711 (1990). 10.1002/ijc.2910460426 1698732

[b5] Tindle R. W., Smith J. A., Geysen H. M., Selvey L. A. & Frazer I. H. Identification of B epitopes in human papillomavirus type 16 E7 open reading frame protein. J Gen Virol. 71, 1347–1354 (1990). 1693666 10.1099/0022-1317-71-6-1347

[b6] Selvey L. A. . Identification of B-epitopes in the human papillomavirus 18 E7 open reading frame protein. J Immunol. 14, 3105–3110 (1990). 1698872

[b7] Christensen N. D. . Surface conformational and linear epitopes on HPV-16 and HPV-18 L1 virus-like particles as defined by monoclonal antibodies. Virology 223, 174–184 (1996). 8806551 10.1006/viro.1996.0466

[b8] Bleul C. . Human papillomavirus type 18 E6 and E7 antibodies in human sera: increased anti-E7 prevalence in cervical cancer patients. J Clin Microbiol. 29, 1579–1588 (1991). 1722219 10.1128/jcm.29.8.1579-1588.1991 PMC270166

[b9] Gao L. . Immune response to human papillomavirus type 16 E6 gene in a live vaccinia vector. J Gen Virol. 75 (Pt 1), 157–164 (1994). 7509369 10.1099/0022-1317-75-1-157

[b10] Heino P. . Human papillomavirus type 16 capsids expose multiple type-restricted and type-common antigenic epitopes. J Gen Virol. 76(Pt 5), 1141–1153 (1995). 7537325 10.1099/0022-1317-76-5-1141

[b11] Combita A. L., Touze A., Bousarghin L., Christensen N. D. & Coursaget P. Identification of two cross-neutralizing linear epitopes within the L1 major capsid protein of human papillomaviruses. J Virol. 76, 6480–6486 (2002). 12050360 10.1128/JVI.76.13.6480-6486.2002 PMC136273

[b12] Christensen N. D., Reed C. A., Cladel N. M., Hall K. & Leiserowitz G. S. Monoclonal antibodies to HPV-6 L1 virus-like particles identify conformational and linear neutralizing epitopes on HPV-11 in addition to type-specific epitopes on HPV-6. Virology. 224, 477–486 (1996). 8874508 10.1006/viro.1996.0554

[b13] Ludmerer S. W., Benincasa D., Mark G. E. & Christensen N. D. A neutralizing epitope of human papillomavirus type 11 is principally described by a continuous set of residues which overlap a distinct linear, surface-exposed epitope. J Virol. 71, 3834–3839 (1997). 9094659 10.1128/jvi.71.5.3834-3839.1997 PMC191534

[b14] Xu W. X. . Minimal motif mapping of a known epitope on human zona pellucida protein-4 using a peptide biosynthesis strategy. J Reprod Immunol. 81, 9–16 (2009). 19539378 10.1016/j.jri.2009.04.004

[b15] Xu W. X. . Mapping of minimal motifs of B-cell epitopes on human zona pellucida glycoprotein-3. Clin Dev Immunol 2012, 831010, doi: 10.1155/2012/831010 (2012). 22162720 PMC3227431

[b16] Zhao R. . Human papillomavirus infection in Beijing, People’s Republic of China: a population-based study. Br J Cancer. 101, 1635–1640 (2009). 19862002 10.1038/sj.bjc.6605351 PMC2778508

[b17] Chan P. K. . Identification of human papillomavirus type 58 lineages and the distribution worldwide. J Infect Dis. 203, 1565–1573 (2011). 21592985 10.1093/infdis/jir157 PMC6281402

[b18] Bouvard V. . A review of human carcinogens—Part B: biological agents. Lancet Oncol, 10, 321–322 (2009). 19350698 10.1016/s1470-2045(09)70096-8

[b19] Liu D. . Fine epitope mapping of the central immunodominant region of nucleoprotein from Crimean-Congo hemorrhagic fever virus (CCHFV). PLoS One. 9(11), e108419 (2014). 25365026 10.1371/journal.pone.0108419 PMC4217714

[b20] Yu R. . Fine mapping and conservation analysis of linear B-cell epitopes of peste des petits ruminants virus nucleoprotein. Vet Microbiol. 175, 132–138 (2015). 25465659 10.1016/j.vetmic.2014.10.012

[b21] Frazer I. H. Immunology of papillomavirus infection. Curr Opin Immunol. 8, 484–491 (1996). 8794007 10.1016/s0952-7915(96)80035-5

[b22] Corti D. . A neutralizing antibody selected from plasma cells that binds to group 1 and group 2 influenza A hemagglutinins. Science. 333, 850–856 (2011). 21798894 10.1126/science.1205669

[b23] Dreyfus C. . Highly conserved protective epitopes on influenza B viruses. Science. 337, 1343–1348 (2012). 22878502 10.1126/science.1222908 PMC3538841

[b24] Geysen H. M., Mason T. J. & Rodda S. J. Cognitive features of continuous antigenic determinants. J Mol Recognit. 1, 32–41 (1988). 2483922 10.1002/jmr.300010107

[b25] Eswar N. . Comparative protein structure modeling using MODELLER. Curr Protoc Protein Sci *Chapter* 2: Unit 2.9 (2007). 10.1002/0471140864.ps0209s50 18429317

[b26] Einstein M. H. . Comparative immunogenicity and safety of human papillomavirus (HPV)-16/18 vaccine and HPV-6/11/16/18 vaccine: follow-up from months 12–24 in Phase III randomized study of healthy women aged 18–45 years. Hum Vaccine. 7, 1343–1358 (2011). 10.4161/hv.7.12.18281 PMC3338932 22048173

[b27] DiMaio F., Leaver-Fay A., Bradley P., Baker D. & André I. Modeling symmetric macromolecular structures in Rosetta3. PLoS One. 6, e20450 (2011). 21731614 10.1371/journal.pone.0020450 PMC3120754

[b28] Xu G. J. . Comprehensive serological profiling of human populations using a synthetic human virome. Science, 348(6239), aaa0698 (2015). 26045439 10.1126/science.aaa0698 PMC4844011

[b29] Wrammert J. . Broadly cross-reactive antibodies dominate the human B cell response against 2009 pandemic HiN1 influenza virus infection. J Exp Med. 208, 181–193 (2011). 21220454 10.1084/jem.20101352 PMC3023136

[b30] Slupetzky K. . A papillomavirus-like particle (VLP) vaccine displaying HPV16 L2 epitopes induces cross-neutralizing antibodies to HPV11. Vaccine 25, 2001–2010 (2007). 17239496 10.1016/j.vaccine.2006.11.049 PMC3935451

[b31] Xu W. X. . Mapping of epitopes relevant for induction of acrosome reaction on human zona pellucida glycoprotein-4 using monoclonal antibodies. Am J Reprod Immunol. 68, 465–475 (2012). 22860757 10.1111/j.1600-0897.2012.01177.x

[b32] Jiang Z., Tong G., Cai B., Xu Y. & Lou J. Purification and immunogenicity study of human papillomavirus 58 virus-like particles expressed in *Pichia pastoris*. Protein Expr Purif. 80, 203–210 (2011). 21821128 10.1016/j.pep.2011.07.009

[b33] Muñoz N. . Epidemiologic classification of human papillomavirus types associated with cervical cancer. N Engl J Med. 348, 518–527 (2003). 12571259 10.1056/NEJMoa021641

[b34] de Sanjose S. . Human papillomavirus genotype attribution in invasive cervical cancer: a retrospective cross-sectional worldwide study. Lancet Oncol. 11, 1048–1056 (2010). 20952254 10.1016/S1470-2045(10)70230-8

[b35] Jacobs M. V. . A general primer GP5+ /GP6(+)-mediated PCR-enzyme immunoassay method for rapid detection of 14 high-risk and 6 low-risk human papillomavirus genotypes in cervical scrapings. J Clin Microbiol. 35, 791–795 (1997). 9041439 10.1128/jcm.35.3.791-795.1997 PMC229677

[b36] Maver P. J., Kocjan B. J., Seme K. & Polja M. Genomic diversity of low-risk human papillomavirus genotypes HPV 40, HPV 42, HPV 43, and HPV 44. J Med Virol. 86, 272–282 (2014). 24155245 10.1002/jmv.23822

[b37] Xin C. Y. . Analysis of E6 variants of human papillomavirus type 33, 52 and 58 in Japanese women with cervical intraepithelial neoplasia/cervical cancer in relation to their oncogenic potential. Cancer Letters. 170, 19–24 (2001). 11448530 10.1016/s0304-3835(01)00570-5

[b38] Chan P. K. . Association of human papillomavirus type 58 variant with the risk of cervical cancer. J Natl Cancer Inst. 94, 1249–1253 (2002). 12189229 10.1093/jnci/94.16.1249

[b39] Chang Y. J. . Unique variants of human papillomavirus genotypes 52 and 58 and risk of cervical neoplasia. J Cancer. 129, 965–973 (2011). 10.1002/ijc.25724 20949622

[b40] Liu J. H. . Variations of human papillomavirus type 58 E6, E7, L1 genes and long control region in strains from women with cervical lesions in Liaoning province, China. Infect Genet Evol. 12, 1466–1472 (2012). 22659102 10.1016/j.meegid.2012.05.004

[b41] Yang L. . Genetic variability of HPV-58 E6 and E7 genes in Southwest China. Infect Genet Evol. 21, 395–400 (2014). 24368255 10.1016/j.meegid.2013.12.009

[b42] de Villiers E. M., Fauquet C., Broker T. R., Bernard H. U. & zur Hausen H. Classification of papillomaviruses. Virology. 324, 17–27 (2004). 15183049 10.1016/j.virol.2004.03.033

[b43] Shi Y. P. . Immunogenicity and *in vitro* protective efficacy of a recombinant multistage *Plasmodium falciparum* candidate vaccine. Proc Natl Acad Sci USA. 96, 1615–1620 (1999). 9990073 10.1073/pnas.96.4.1615 PMC15536

[b44] He Y. P. . Immunogenic comparison for two different recombinant chimeric peptides (CP12 and CP22) containing one or two copies of three linear B cell epitopes from β-hCG subunit. J Biotechnol. 151, 15–21 (2011). 21084058 10.1016/j.jbiotec.2010.11.003

[b45] Scarselli M. . Rational design of a meningococcal antigen inducing broad protective immunity. Sci Transl Med. 3, 91–62 (2011). 10.1126/scitranslmed.3002234 21753121

[b46] Zhao Z. . Multiple B-cell epitope vaccine induces a Staphylococcus enterotoxin B-specific IgG1 protective response against MRSA infection. Sci Rep. 5, 12371, doi: 10.1038/srep12371 (2015). 26201558 PMC4511869

[b47] Slepushkin V. A. . Protection of mice against influenza challenge by vaccination with baculovirus-expressed M2 protein. Vaccine. 13, 1399–1402 (1995). 8578816 10.1016/0264-410x(95)92777-y

[b48] Neirynck S. . A universal influenza A vaccine based on the extracellular domain of the M2 protein. Nat Med. 5, 1157–1163 (1999). 10502819 10.1038/13484

[b49] Serrano B. . Potential impact of a 9-valent HPV vaccine in HPV-related cervical disease in 4 emerging countries (Brazil, Mexico, India and China). Cancer Epidemiol. 38, 748–756 (2014). 25305098 10.1016/j.canep.2014.09.003

[b50] Ishizaki A. . E6 and E7 variants of human papillomavirus-16 and -52 in Japan, the Philippines, and Vietnam. J Med Virol. 85, 1069–1076 (2013). 23588734 10.1002/jmv.23566

[b51] Robbins J. B., Schneerson R. & Szu S. C. Perspective hypothesis: serum IgG antibody is sufficient to confer protection against infectious diseases by inactivating the inoculum. J Infect Dis. 171, 1387–1398 (1995). 7769272 10.1093/infdis/171.6.1387

[b52] Christensen N. D. . Human papillomavirus types 6 and 11 have antigenically distinct strongly immunogenic conformationally dependent neutralizing epitopes. Virology. 205, 329–335 (1994). 7526536 10.1006/viro.1994.1649

[b53] Hiller T., Poppelreuther S., Stubenrauch F. & Iftner T. Comparative analysis of 19 genital human papillomavirus types with regard to p53 degradation, immortalization, phylogeny, and epidemiologic risk classification. Cancer Epidemiol Biomarkers Prev. 15, 1262–1267 (2006). 16835321 10.1158/1055-9965.EPI-05-0778

[b54] Wong F. K., Ching J. C. & Chow J. K. Comparison of the DiagCor GenoFlow Human Papillomavirus Array Test and Roche Linear Array HPV Genotyping Test. Open Virol J. 4, 169–174 (2010). 21270940 10.2174/1874357901004010169 PMC3026337

[b55] Ho G. Y., Bierman R., Beardsley L., Chang C. J. & Burk R. D. Natural history of cervicovaginal papillomavirus infection in young women. N Engl J Med. 338, 423–428 (1998). 9459645 10.1056/NEJM199802123380703

[b56] Saito M., Okubo M., Hirata R., Takeda S. & Maeda H. Association of human leukocyte antigen and T cell message with human papillomavirus 16-positive cervical neoplasia in Japanese women. Int J Gynecol Cancer. 17, 1314–1321 (2007). 17433060 10.1111/j.1525-1438.2007.00938.x

[b57] Kirii Y., Iwamoto S. & Matsukura T. Human papillomavirus type 58 DNA sequence. Virology. 185, 424–427 (1991). 1656594 10.1016/0042-6822(91)90791-9

[b58] Zou Y. S., Xu W. X., Sun Z. D. & Xue X. L. Preparation of polyacrylamide gel electrophoresis for human chorionic gonadotropin chimeric peptide 12 expressed in *E. coli*. Acta Biochim Biophys Sin. 34, 671–674 (2002). 12198575

