# Supplementary material for: Epitomics: IgG-epitome decoding of E6, E7 and L1 proteins from oncogenic human papillomavirus type 58
Source: Sci Rep. 2016 Oct 6;6:34686. doi: 10.1038/srep34686 (PMC5052575; doi:10.1038/srep34686)

Epitomics: IgG-epitome decoding of E6, E7 and L1 proteins from oncogenic human papillomavirus type 58

Wan-Xiang Xu, Jian Wang, Hai-Ping Tang, Ya-Ping He, Qian-Xi Zhu, Satish K. Gupta, Shao-Hua Gu, Qiang Huang, Chao-Neng Ji, Ling-Feng Liu, Gui-Ling Li, Cong-Jian Xu and Yi Xie

Figure S1 Recombinant E6, E7, L1N and L1C proteins. (**A-D**) SDS-PAGE analysis of expressed and purified E6, L1N (aa residues 36-234), L1C (aa residues 227-524), and E7; (**E**) Western blotting of expressed and purified E7 with mAb against His$_6$-tag. Lanes 1, 6, 11, and 16, pre-stained protein markers; lanes 2, 7, 12, and 17/21, uninduced cell proteins; lanes 3, 8, 13, and 18/22 induced cell proteins; lanes 4, 9, 14, and 19/23, 2 μg of purified E6, L1N, L1C, and E7 proteins; lanes 5, 10, 15, and 20, 2 μg bovine serum albumin.

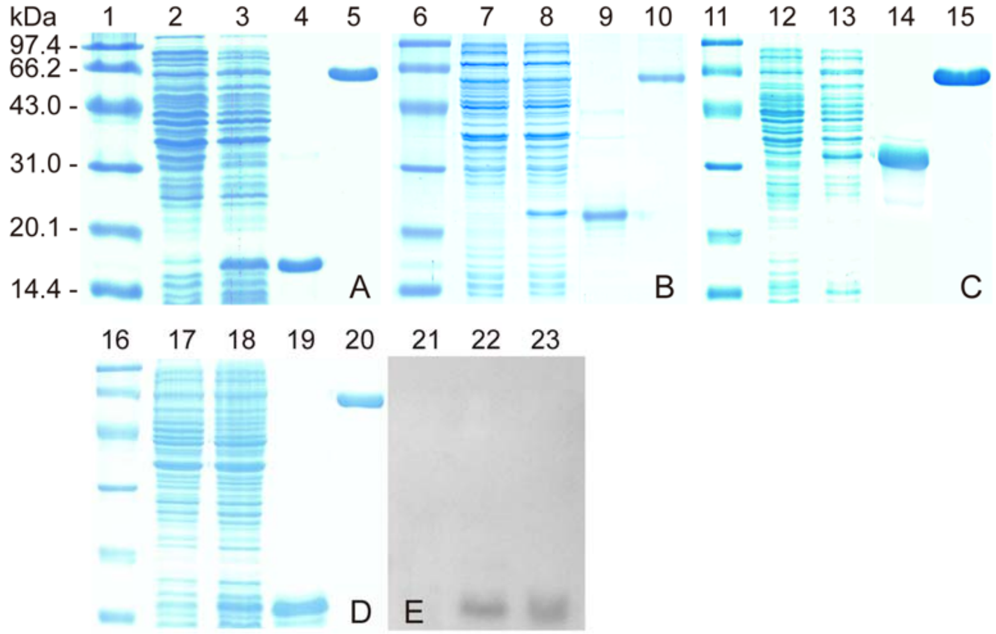

Figure S2 Comparison of antibody titers to r-E6 (A), r-E7 (B), L1N (C) and L1C (D) in the group of immunized rabbits. The antibody levels were determined by ELISA using the respective r-proteins as antigens. In each sub-panel, different colored lines represent (except blue line represents serum from rabbit immunized with adjuvant alone as negative control) individual immunized rabbits. Titers were determined based on the highest dilution of the sample that generated OD greater than 0.2.

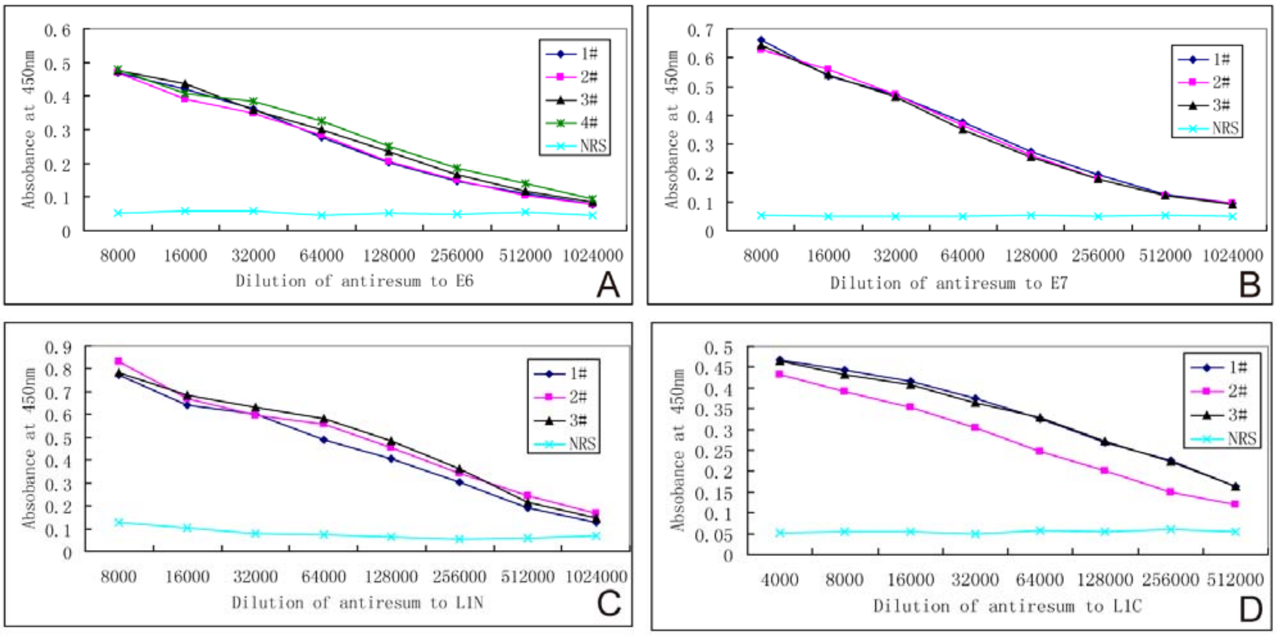

Table S1 Analysis of the common sequence in reactive 6-8mer peptides for mapping BCEs of E7

Peptide NO.	Amino acid Sequence	position in HPV58 E7	Peptide NO.	Amino acid sequence	position in HPV58 E7
P16	MRGNNPTL	1-8	P50	DEIGLDGP	35-42
P17	RGNNPTLR	2-9	P51	EIGLDGPD	36-43
P18	GNNPTLRE	3-10	P52	IGLDGPDG	37-44
P19	NNPTLREY	4-11	P53	GLDGPDGQ	38-45
P20	NPTLREYI	5-12	P54	LDGPDGQA	39-46
P21	PTLREYIL	6-13	P55	DGPDGQAQ	40-47
P22	TLREYILD	7-14	P56	GPDGQAQP	41-48
P23	LREYILDL	8-15	P57	PDGQAQPA	42-49
P24	REYILDLH	9-16	P58	DGQAQPAT	43-50
P25	EYILDLHP	10-17	P59	GQAQPATA	44-51
P26	YILDLHPE	11-18	P60	MGTCTIVC	85-92
P27	ILDLHPEP	12-19	P61	GTCTIVCP	86-93
P28	LDLHPEPT	13-20	P62	TCTIVCPS	87-94
P29	DLHPEPTD	14-21	P63	CTIVCPSC	88-95
P30	LHPEPTDL	15-22	P64	TIVCPSCA	89-96
P31	HPEPTDLF	16-23	P65	IVCPSCAQ	90-97
P32	PEPTDLFC	17-24	P66	VCPSCAQQ	91-98
P33	EPTDLFCY	18-25	P67	CPSCAQQ	92-98
P34	PTDLFCYE	19-26	P68	PSCAQQ	93-98
P35	TDLFCYEQ	20-27	P69	A-SCAQQ	94-98
P36	DLFCYEQL	21-28	P70	AA-CAQQ	95-98
P37	LFCYEQLC	22-29	P71	AAA-AQQ	96-98
P38	FCYEQLCD	23-30	P72	AAA-DSSDED	30-35
P39	CYEQLCDS	24-31	P73	AAA- SSDED	31-35
P40	YEQLCDSS	25-32	P74	AAA- SDED	32-35
P41	EQLCDSSD	26-33	P75	AAA- DED	33-35
P42	QLCDSSDE	27-34	P76	AAA-EIG	36-38
P43	LCDSSDED	28-35	P77	AAA-EIGL	36-39
P44	CDSSDEDE	29-36	P78	AAA-EIGLD	36-40
P45	DSSDEDEI	30-37	P79	AA-EDEIGL	34-39
P46	SSDEDEIG	31-38	P80	A-DEDEIGL	33-39
P47	SDEDEIGL	32-39	P47	SDEDEIGL	32-39
P48	DEDEIGLD	33-40	P81	SDEDEIG-A	32-38
P49	EDEIGLDG	34-41	P82	SDEDEI-AA	32-37

The cyan highlight indicates minimal motif of each epitope (E7-1 to E7-9); the yellow indicates those of E7-5 and E7-6 mapped by 7 deduced fine epitope peptides; and the green indicates another E7-9 epitope mapped, which was not counted as an epitope in E7-epitome due to its position nested between conjoint sequences of E7-5 and E7-6.

Table S2. Analysis of the common sequence in reactive 8mer peptides for mapping BCEs of L1

NO.	Peptide sequence	Range	NO.	Peptide sequence	Range
P61	MTVYLPPV	35-42	P101	NRYPAQPG	160-167
P62	TVYLPPVP	36-43	P102	RYPAQPGS	161-168
P63	VYLPPVPV	37-44	P103	YPAQPGSD	162-169
P64	YLPPVPVS	38-45	P104	PAQPGSDN	163-170
P65	LPPVPVSK	39-46	P105	AQPGSDNR	164-171
P66	PPVPVSKV	40-47	P106	QPGSDNRE	165-172
P67	PVPVSKVV	41-48	P107	NNNAAATD	203-210
P68	VPVSKVVS	42-49	P108	NNAAATDC	204-211
P69	PVSKVVST	43-50	P109	NAAATDCP	205-212
P70	VSKVVSTD	44-51	P110	AAATDCPP	206-213
P71	SKVVSTDE	45-52	P111	AATDCPPL	207-214
P72	VFRVRLPD	99-106	P112	ATDCPPLE	208-215
P73	FRVRLPDP	100-107	P113	TDCPPLEL	209-216
P74	RVRLPDPN	101-108	P114	DCPPLELF	210-217
P75	VRLPDPNK	102-109	P115	CPPLELFN	211-217
P76	RLPDPNKF	103-110	P116	PPLELFNS	212-218
P77	LPDPNKFG	104-111	P117	PLELFNSI	213-219
P78	PDPNKFGF	105-112	P118	LELFNSII	214-220
P79	DPNKFGFP	106-113	P119	SIIEDGDM	219-226
P80	PNKFGFPD	107-114	P120	IIEDGDMV	220-227
P81	NKFGFPDT	108-115	P121	IEDGDMVD	221-228
P82	KFGFPDTS	109-116	P122	EDGDMVDT	222-229
P83	VGVSGHPY	142-149	P123	DGDMVDTG	223-230
P84	GVSGHPYL	143-150	P124	GDMVDTGF	224-231
P85	VSGHPYLN	144-151	P125	DMVDTGFG	225-232
P86	SGHPYLNK	145-152	P126	MVDTGFGC	226-233
P87	GHPYLNKF	146-153	P127	VDTGFGCM	227-234
P88	HPYLNKFD	147-154	P128	DTGFGCMD	228-235
P89	PYLNKFDD	148-155	P129	TGFGCMDF	229-236
P90	YLNKFDDT	149-156	P130	GFGCMDFG	230-237
P91	LNKFDDTE	150-157	P131	FGCMDFGT	231-238
P92	NKFDDTET	151-158	P132	GCMDFGTL	232-239
P93	KFDDTETS	152-159	P133	CMDFGTLQ	233-240
P94	FDDTETSN	153-160	P134	MDFGTLQA	234-241
P95	DDTETSNR	154-161	P135	DFGTLQAN	235-242
P96	DTETSNRY	155-162	P136	FGTLQANK	236-243
P97	TETSNRYP	156-163	P137	GTLQANKS	237-244
P98	ETSNRYPA	157-164	P138	TLQANKSD	238-245
P99	TSNRYPAQ	158-165	P139	LQANKSDV	239-246
P100	SNRYPAQP	159-166	P140	QANKSDVP	240-247

P141	ANKSDVPI	241-248	P182	EGTYKNDN	377-384
P142	NKSDVPID	242-249	P183	GTYKNDNF	378-385
P143	KSDVPIDI	243-250	P184	TYKNDNFK	379-386
P144	SDVPIDIC	244-251	P185	YKNDNFKE	380-387
P145	DVPIDICN	245-252	P186	KNDNFKEY	381-388
P146	VPIDICNS	246-253	P187	NDNFKEYV	382-389
P147	PIDICNST	247-254	P188	DNFKEYVR	383-390
P148	IDICNSTC	248-255	P189	NFKEYVRH	384-391
P149	DICNSTCK	249-256	P190	FKEYVRHV	385-392
P150	ICNSTCKY	250-257	P191	KEYVRHVE	386-393
P151	CNSTCKYP	251-258	P192	EYVRHVEE	387-394
P152	NSTCKYPD	252-259	P193	YVRHVEEY	388-395
P153	AGKLGEAV	290-297	P194	AITCQKTA	450-457
P154	GKLGEAVP	291-298	P195	ITCQKTAP	451-458
P155	KLGEAVPD	292-299	P196	TCQKTAPP	452-459
P156	LGEAVPDD	293-300	P197	CQKTAPPK	453-460
P157	GEAVPDDL	294-301	P198	QKTAPPKE	454-461
P158	EAVPDDLY	295-302	P199	KTAPPKEK	455-462
P159	AVPDDLYI	296-303	P200	TAPPKEKE	456-463
P160	VPDDLYIK	297-304	P201	APPKEKED	457-464
P161	PDDLYIKG	298-305	P202	PPKEKEDP	458-465
P162	DDLYIKGS	299-306	P203	PKEKEDPL	459-466
P163	DLYIKGSG	300-307	P204	KEKEDPLN	460-467
P164	SGSIVTSE	322-329	P205	EKEDPLNK	461-468
P165	GSIVTSES	323-330	P206	KEDPLNKY	462-469
P166	SIVTSESQ	324-331	P207	EDPLNKYT	463-470
P167	IVTSESQL	325-332	P208	DPLNKYTF	464-471
P168	VTSESQLF	326-333	P209	PLNKYTFW	465-472
P169	TSESQLFN	327-334	P210	GLKAKPRL	498-505
P170	SESQLFNK	328-335	P211	LKAKPRLK	499-506
P171	ESQLFNKP	329-336	P212	KAKPRLKR	500-507
P172	SQLFNKPY	330-337	P213	AKPRLKRS	501-508
P173	QLFNKPYW	331-338	P214	KPRLKRSA	502-509
P174	LFNKPYWL	332-339	P215	PRLKRSAP	503-510
P175	LCTEVTKE	370-377	P216	RLKRSAPT	504-511
P176	CTEVTKEG	371-378	P217	LKRSAPTT	505-512
P177	TEVTKEGT	372-379	P218	KRSAPTTR	506-513
P178	EVTKEGTY	373-380	P219	RSAPTTRA	507-514
P179	VTKEGTYK	374-381	P220	SAPTTRAP	508-515
P180	TKEGTYKN	375-382	P221	APTTRAPS	509-516
P181	KEGTYKND	376-383			

The cyan highlight indicates minimal motif of each epitope (L1-1 to L1-18).

Table S3. Conservative analysis of HPV58-BCE motifs among LR-HPVs and other unknown LR-/HR-HPVs

HPV58	HPV types and aligning sequence (aa range)																	
BCE No.	6*	11*	40*	42*	43*	44*	30	34	54	61	62	67	69	70	72	81	85	89	91
E6-2								85-89				84-88	84-88				86-90		
E6-3								123-127											
E7-2	12-15	12-15						12-15	12-15			12-15	12-15	12-15			12-15		
L1-2					78-83			105-110				110-115							170-175
L1-3					117-122														209-214
L1-4				153-156	125-128		134-137		124-127	129-132	127-130	157-160	125-128	127-130	128-131	128-131	127-130	144-147	
L1-7	183-187	184-188		211-215		184-188	194-198	210-214	183-187			217-221	187-191	185-189			187-191		
L1-8						193-198												212-217	286-291
L1-9	198-201		200-203		200-203	199-202						232-235	202-205						292-295
L1-11								242-246											
L1-12		269-272						295-298											
L1-13	301-305	316-320		332-336	303-307	301-305	312-316			306-310	306-310	335-339	306-310	303-307	307-311	307-311		323-327	395-399
L1-16							443-446		431-433			466-469	439-442		440-443	439-442			528-531
L1-18					492-494		492-494	511-513			487-489								583-585

Asterisk indicates the widely accepted typical LR-HPV types. Other unknown or contentious HR- or LR-HPVs are from refs. 33, 34, and 59-61.

Table S4. Analysis of mutations of amino acids related with E6-2 in HPV16, 18 and 52 variants

HPV Types	Countries/ Regions	Refs.	Mutation of amino acids and their position								D/X mutation /variant numbers
16	Worldwide	62	K11Q	L12V	H24Y	D25N	E30Q	A61G	R77K		0/45
	Sweden	63	Q3E	R10G	K11T	Q14H	D25N	I27R	N29S	H51N	0/14
			R55K	N58S	A61G	H78Y	L83V				
	Italy	64	Q14H	Q20H	E29G	K34Q	K65T	K72N	H78Y	L83V	0/16
			F125C	N127H	G134D	C136R	C140S	S142P			
	Northeast, China	65	D25N	D25E	N58S	C80G	L83V	E113D			0/30
	Liaoning, China	66	R10G	D25E	H78Y	L83V	E113D				0/199
	Taiwan	67	R10G	K11Q	K11T	L12V	Q14H	D25Q	D25E	I27R	0/152
			L38P	I52M	N58S	N58K	H78Y	L83V	E113D		
	Japan, Philippines and Vietnam	50	R10I	Q14D	D25E	R48W	I52V	H78Y	L83V	E113D	0/62
			R141T								
18	Indonesia	68	C105W	N129K							0/4
	Portugal	69	H80Y	N129K							0/16
	Mexico	70	H80Y	N129K							0/2
	Northeast, China	71	H131N								0/2
52	Japan	37	K93R								0/45
	Taiwan	39	K34R	K93G	K93R	S97R	E116K	N122K			0/235
	Northeast, China	72	K93R								0/65
	Japan, Philippines and Vietnam	50	H24D	K93R	K93G	N122K					0/85

Supplementary references

59. Hwang, T. S. *et al*. Detection and typing of HPV genotypes in various cervical lesions by HPV oligonucleotide microarray. *Gynecol Oncol*. **90**, 51-56 (2003).

60. Dockter, J. *et al*. Clinical performance of the APTIMA HPV Assay for the detection of high-risk HPV and high-grade cervical lesions. *J Clin Virol*. **45** (Suppl 1), S55-61 (2009).

61. Chen, Z. *et al*. Evolution and taxonomic classification of alphapapillomavirus 7 complete genomes: HPV18, HPV39, HPV45, HPV59, HPV68 and HPV70. *PLoS One*. **8**, e72565 (2013).

62. Yamada, T. *et al*. Human papillomavirus type 16 sequence variation in cervical cancers: a worldwide perspective. *J Virol*. **71**, 2463-2472 (1997).

63. Zehbe, I. Wilander, E., Delius, H. & Tommasino, M. Human papillomavirus 16 E6 variants are more prevalent in invasive cervical carcinoma than the prototype. *Cancer Res*. **58**, 829-833 (1998).

64. Cento, V., Ciccozzi, M., Ronga, L., Perno, C. F. & Ciotti, M. Genetic diversity of human papillomavirus type 16 E6, E7 and L1 genes in Italian women with different grades of cervical lesions. *J Med Virol*. **81**, 1627-1634 (2009).

65. Shang, Q. *et al*. Human papillomavirus type 16 variant analysis of E6, E7, and L1 [corrected] genes and long control region in [corrected] cervical carcinomas in patients in northeast China. *J Clin Microbiol*. **49**, 2656-2663 (2011).

66. Sun, Z. *et al*. Genetic variations of E6 and long control region of human papillomavirus type 16 from patients with cervical lesion in Liaoning, China. *BMC Cancer*. **13**, 459 (2013).

67. Chang, Y. J. *et al*. Intratypic variants of human papillomavirus type 16 and risk of cervical neoplasia in Taiwan. *J Med Virol*. **85**, 1567-1576 (2013).

68. De Boer, M. A. *et al*. 2005. Human papillomavirus type 18 variants: histopathology and E6/E7 polymorphisms in three countries. *Int J Cancer*. **114**, 422-425 (2005).

69. De la Cruz-Hernández, E. *et al*. 2005. Differential splicing of E6 within human papillomavirus type 18 variants and functional consequences. *J Gen Virol*. **86**(Pt 9), 2459-68 (2005).

70. Pista, A. *et al*. 2007. Molecular variants of human papillomavirus type 16 and 18 and risk for cervical neoplasia in Portugal. *J Med Virol*. **79**, 1889-1897 (2007).

71. Sun, Z. *et al*. Variant lineages of human papillomavirus type 18 in Northeast China populations characterized by sequence analysis of E6, E7, and L1 regions. *Int J Gynecol Cancer*. **22**, 930-936 (2012).

72. Sun, Z. *et al*. Genomic polymorphism of human papillomavirus type 52 in women from Northeast China. *Int J Mol Sci*. 13, 14962-14972 (2012).

The full length figures of SDS-PAGE gels and blots:

Figure S3 for Figure 1

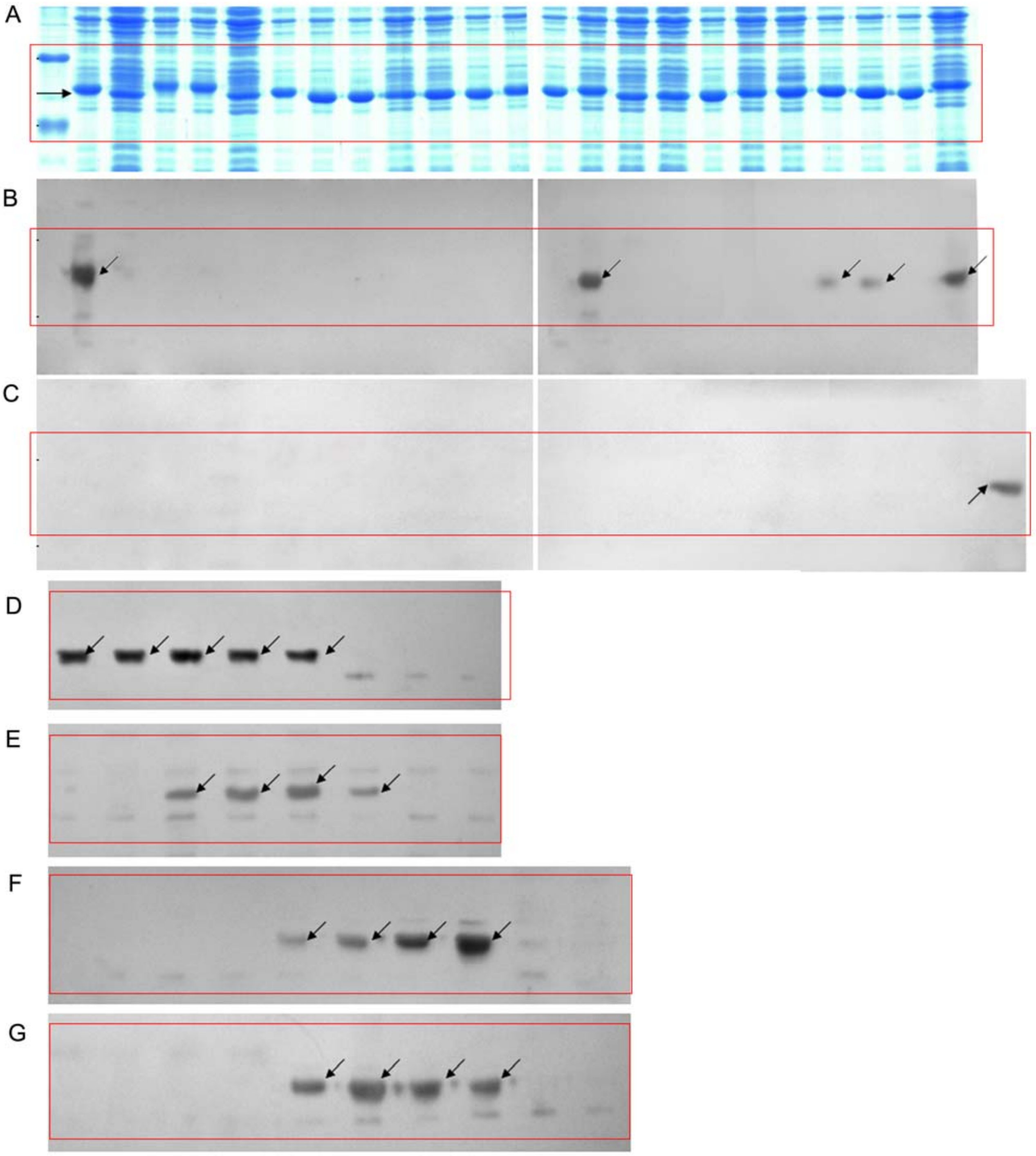

Figure S4 for Figure 2

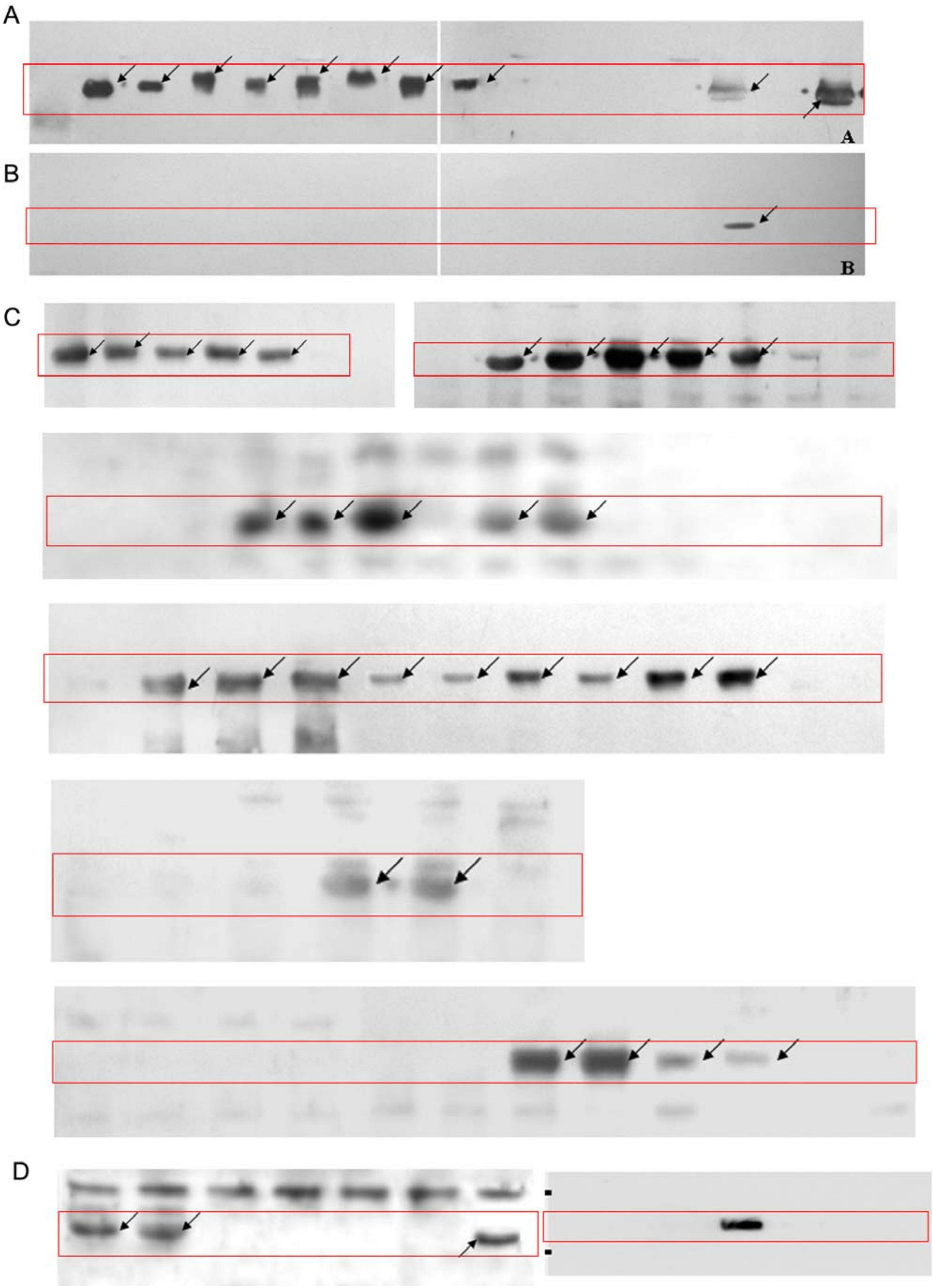

Figure S5 for Figure 3

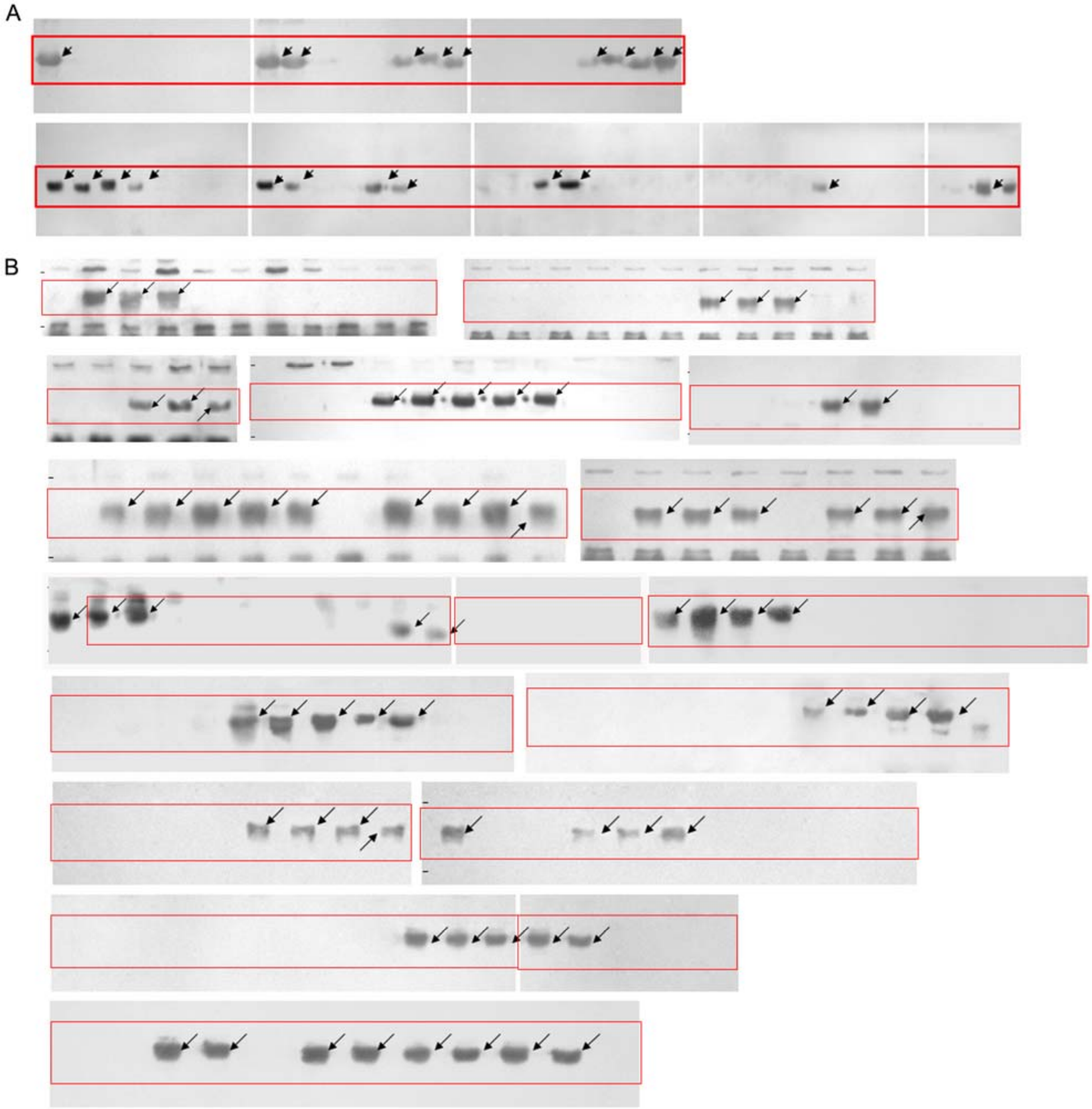

Figure S6 for Figure 4

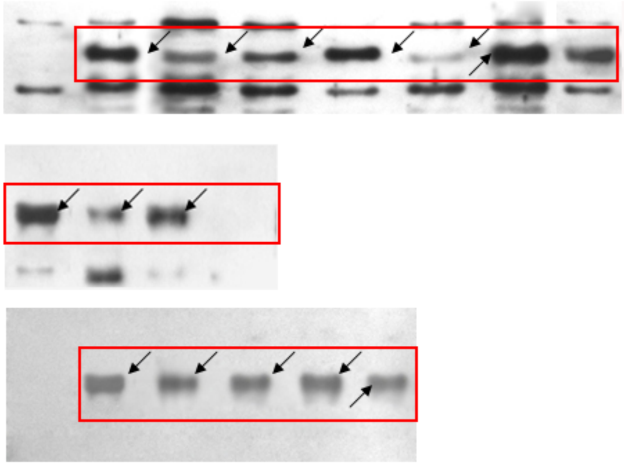

Figure S7 for Figure 5

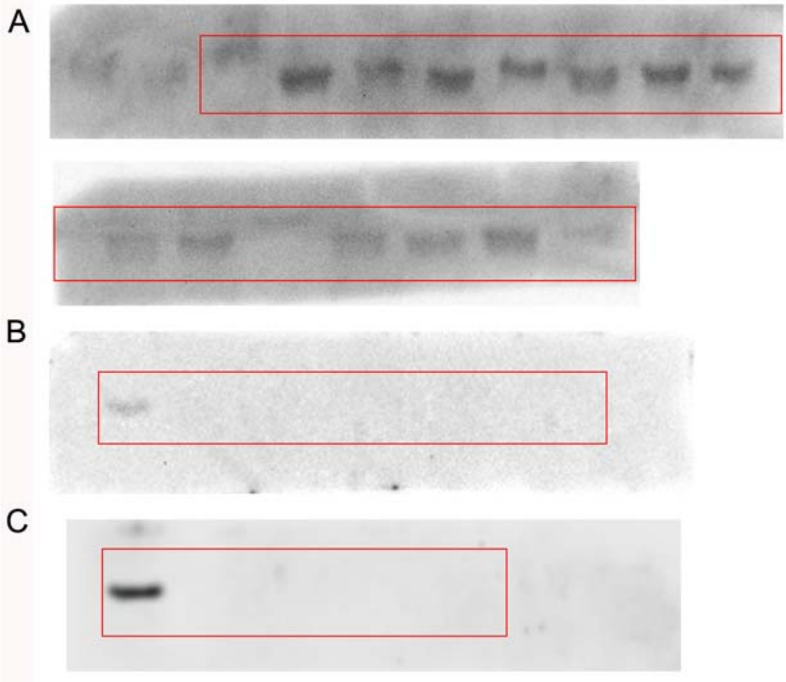

Supplement: Supplementary Information [file srep34686-s1.pdf]
